# Biomarker-based approach to human exposure assessment of furan in food

**DOI:** 10.1007/s00204-025-04022-2

**Published:** 2025-04-04

**Authors:** C. Kalisch, M. Reiter, A. Mally

**Affiliations:** Department of Toxicology, University of Würzburg, Versbacher Str. 9, 97078 Würzburg, Germany

**Keywords:** Furan, Process-related food contaminant, Human biomonitoring, Urinary biomarker of exposure, Exposure assessment

## Abstract

Humans are continuously exposed to furan, a hepatotoxic and carcinogenic process-related food contaminant. Considerable uncertainties associated with current exposure estimates based on the content of furan in food underscore the need to explore biomarker monitoring as an alternative or complementary approach to furan exposure assessment. Previous work in rats demonstrated a linear correlation between furan dose and several urinary furan metabolites, whereby *N*-[4-carboxy-4-(3-mercapto-1*H*-pyrrol-1-yl)-1-oxobutyl]-L-cysteinylglycine (GSH-BDA), the glutathione (GSH) conjugate of the reactive furan metabolite *cis*-2-butene-1,4-dial (BDA), was identified as a specific biomarker of exogenous furan exposure. To further test the validity of GSH-BDA as a biomarker of furan exposure, the present study was designed to monitor urinary excretion of furan metabolites in human volunteers after consumption of diets with low vs. high furan content using stable isotope dilution LC–MS/MS, and to investigate if analysis of GSH-BDA in human urine is suitable for translating biomarker levels into probable daily intakes. Ten healthy volunteers (*n* = 5/sex) consumed a low-furan diet for three days (day 1–3), followed by consumption of foods with high furan content for three days (day 4–6) and returned to a low-furan diet for a further three days (7–9). Urinary GSH-BDA excretion significantly increased during periods of a high-furan diet, and rapidly declined upon returning to a low-furan diet. Probable daily intakes estimated from GSH-BDA excretion in non-smoking subjects and excretion rates previously determined in F344/DuCrl rats ranged from 0.05 to 0.31 µg/kg bw/d during periods of low-furan diet and increased to 0.18–1.20 µg/kg bw/d during consumption of a high-furan diet. These estimates are in good agreement with exposures reported by the European Food Safety Authority (EFSA), which range between 0.11 to 0.75 µg/kg bw per day (minimum LB to maximum UB) for the average adult and 0.20 to 1.22 µg/kg bw per day (minimum LB to maximum UB) for highly exposed adult consumers (95th percentile). Interestingly, higher excretion of GSH-BDA was observed in smoking compared to non-smoking individuals, highlighting tobacco smoke as a significant source of furan exposure and confounding factor when estimating furan exposure via diet. In contrast to GSH-BDA, high urinary background levels of lysine adducts of BDA and BDA-derived cysteine-lysine crosslinks limit their suitability as biomarkers of exogenous furan exposure.

**Supplementary Information:**

The online version contains supplementary material available at 10.1007/s00204-025-04022-2.

## Introduction

In its 2017 risk assessment of furan in food, the European Food Safety Authority (EFSA) concluded that dietary exposure to furan, a hepatotoxic and -carcinogenic process contaminant, presents a health concern (EFSA 2017). For risk characterization, EFSA used the margin of exposure (MOE) approach applied to neoplastic and non-neoplastic lesions in rodents and human exposures estimated from occurrence data of furan in food and European dietary surveys. For the calculation of MOEs, the Benchmark dose lower confidence limits of 10% of 0.064 mg/kg bw per day for non-neoplastic effects (cholangiofibrosis in male rats) and 1.31 mg/kg bw per day for neoplastic effects (hepatocellular adenomas and carcinomas in female mice) were used as reference points. The MOEs for non-neoplastic and neoplastic effects were below 100, respectively 10,000, in a number of dietary surveys. This indicates that current human exposure to furan from food is of concern, particularly in highly exposed infants and adult consumers, and emphasizes the need for mitigation (EFSA 2017).

Exposure assessment of furan based on the concentration of furan in food is, however, subject to uncertainty, which may lead to over- or underestimation of furan exposure. Main sources of uncertainty include extrapolation of occurrence data to the whole of Europe, individual consumer preparation preferences, exclusion of home-cooking practices, assumptions regarding the contribution of commercially processed foods to the consumption, as well as evaporation loss during reheating of commercially processed foods (EFSA 2017). Thus, alternative or complementary approaches are needed for a more reliable assessment of human furan exposure via food.

**Fig. 1:**
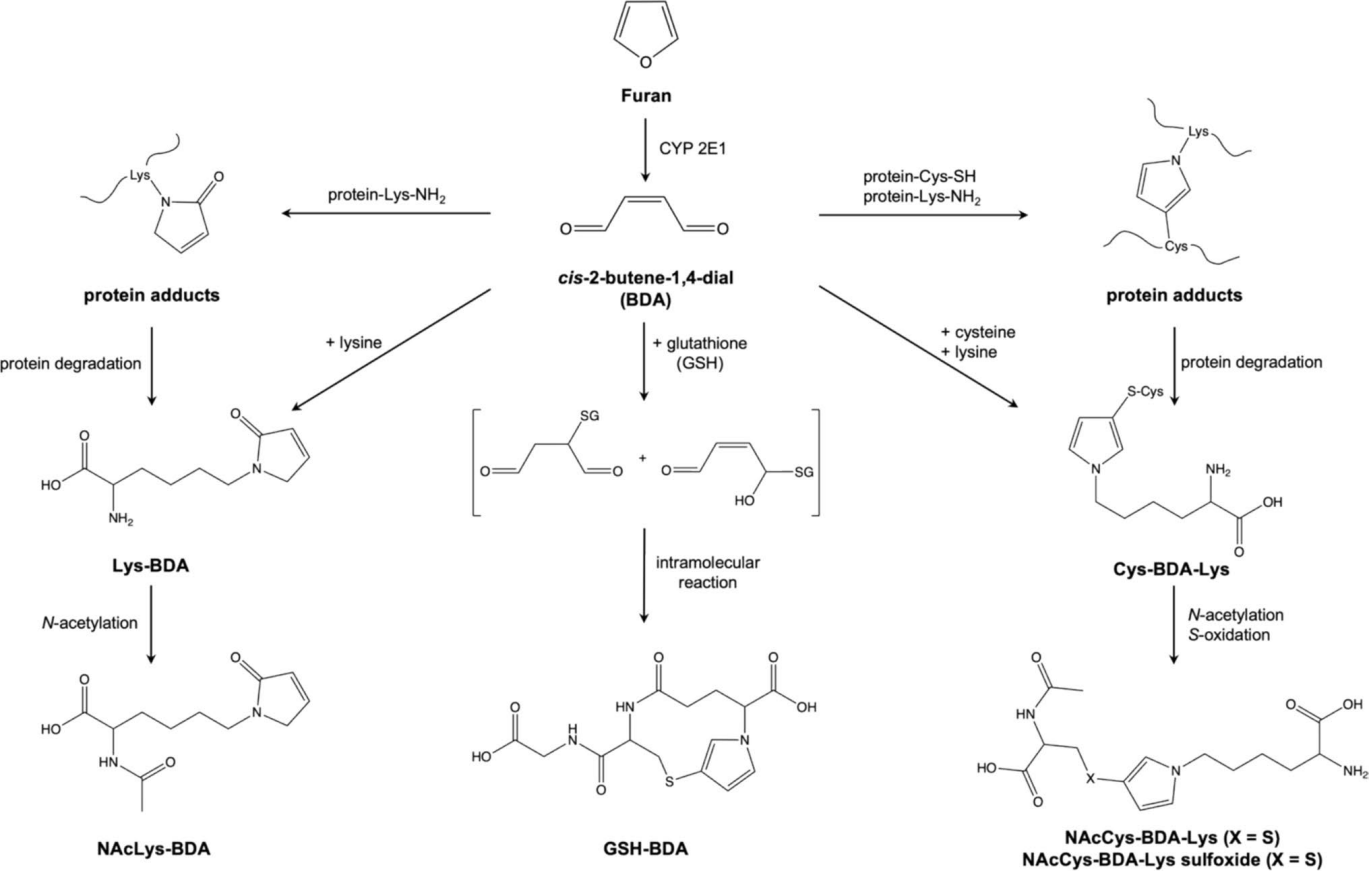
Proposed metabolic pathways of furan in humans, showing urinary furan-derived metabolites as potential biomarkers of furan exposure. Furan is metabolized by CYP 2E1 to the highly reactive dialdehyde *cis*-2-butene-1,4-dial (BDA), which subsequently reacts with cellular nucleophiles (e.g. glutathione, lysine, cysteine). GSH-BDA results from the conjugation of BDA with GSH, followed by intramolecular reaction to the cyclic adduct GSH-BDA. NAcLys-BDA is the product of the conjugation of BDA with lysine and subsequent acetylation of the *⍺*-amino group of lysine. NAcCys-BDA-Lys and the corresponding sulfoxide, NAcCys-BDA-Lys sulfoxide, arise from the crosslink reaction of BDA with cysteine and lysine, with subsequent *N*-acetylation and *S*-oxidation. Conjugation of BDA with GSH and cysteine may also occur in position 2 instead of 3 of the pyrrole structure

Biomarker monitoring has been proposed as a promising approach for assessing human exposure to process-related food contaminants, including furan (Rietjens et al. 2018). Bioactivation of furan via CYP2E1 to the highly reactive intermediate *cis*-2-butene-1,4-dial (BDA) and subsequent reaction with glutathione (GSH) and free or protein-bound lysine and cysteine residues gives rise to a broad spectrum of furan metabolites, which may serve as biomarkers of furan exposure (Fig. 1) (Kellert et al. 2008; Lu et al. 2009; Lu and Peterson 2010; Rietjens et al. 2018; Karlstetter and Mally 2020).

A recent study designed to validate putative biomarkers of furan exposure through quantitative analysis of furan metabolites in urine of male and female F344 rats exposed to isotopically labeled [^13^C_4_]-furan demonstrated a linear correlation between external dose and urinary excretion of the furan metabolites GSH-[^13^C_4_]-BDA (*N*-[4-carboxy-4-(3-mercapto-1*H*-pyrrol-1-yl)-1-oxobutyl]-L-cysteinylglycine), NAcLys-[^13^C_4_]-BDA (*R*-2-(acetylamino)-6-(2,5-dihydro-2-oxo-1*H*-pyrrol-1-yl)-1-hexanoic acid), NAcCys-[^13^C_4_]-BDA-NAcLys (*N*-acetyl-S-[1-[5-(acetylamino)-5-carboxypentyl]-1*H*-pyrrol-3-yl]-L-cysteine) and NAcCys-[^13^C_4_]-BDA-NAcLys sulfoxide (*N*-acetyl-S-[1-[5-(acetylamino)-5-carboxypentyl]-1*H*-pyrrol-3-yl]-L-cysteine sulfoxide) across a wide dose-range (Kalisch et al. 2024). Although 24h-excretion rates of the individual metabolites were low (< 2.5% of the external dose), the close correlation between furan dose and biomarker excretion generally supported the potential of a biomarker-based approach to estimate human furan exposure from food. However, simultaneous analysis of unlabeled metabolites revealed a high and fairly constant background of unlabeled NAcLys-BDA, NAcCys-BDA-NAcLys and NAcCys-BDA-NAcLys sulfoxide in rat urine, which by far exceeded excretion of [^13^C_4_]-furan derived metabolites at dose levels relevant to daily human exposures. As animal feed was shown not to contain significant levels of furan (< 5 µg/kg), the source of this endogenous or exogenous background remains unknown. GSH-BDA was identified as the only metabolite without background occurrence, highlighting its potential as a specific biomarker of external furan exposure (Kalisch et al. 2024). Besides studies in animals, there are as yet only a few studies in humans analyzing furan metabolites as potential biomarkers, focusing on furan exposure via smoking and coffee consumption (Grill et al. 2015; Kremer et al. 2023; Vevang et al. 2023).

With the overall aim of further testing the validity of a biomarker-based approach for human exposure assessment, the aim of the present study was to monitor urinary excretion of furan metabolites in human volunteers after consumption of diets with low vs. high furan content using stable isotope dilution LC–MS/MS, and to investigate if analysis of GSH-BDA in human urine is suitable for translating biomarker levels into probable daily intakes. Analysis of urinary caffeic acid and cotinine was included to assess the impact of coffee and cigarette consumption as major dietary and non-dietary sources of furan exposure on furan metabolite excretion.

## Material and methods

### Chemicals and reagents

Standardized cotinine solution (1.0 mg/mL in methanol) and cotinine-methyl-d_3_ were purchased from Cerilliant (Round Rock, Texas). Caffeic acid and β-glucuronidase type H-2 were from Sigma-Aldrich. HPLC-grade acetonitrile, LC–MS grade methanol and LC–MS grade water were obtained from Roth (Karlsruhe, Germany). LC–MS grade formic acid was from Thermo Fischer Scientific (Waltham, MA). Syntheses of reference substances (GSH-BDA, NAcLys-BDA, NAcCys-BDA-NAcLys, NAcCys-BDA-NAcLys sulfoxide) and internal standards (NAc-[^13^C_6_^15^N_2_]-Lys-BDA, [^13^C_2_]-NAcCys-BDA-NAcLys, [^13^C_2_]-NAcCys-BDA-NAcLys sulfoxide) were performed as previously described (Kalisch et al. 2024). [^13^C_2_^15^N_2_]-GSH-BDA was synthesized as previously published (Karlstetter and Mally 2020). All other chemicals were from Merck (Darmstadt, Germany) or Roth (Karlsruhe, Germany) unless stated otherwise.

### Human study

#### Subjects

The human proof-of-concept study was approved by the ethical committee of the University of Würzburg and was performed according to the Declaration of Helsinki. Ten healthy volunteers (five males and five females; age 24–55; body weight 55.0–87.3 kg) were chosen for this study based on the following inclusion criteria: age between 20 and 60 years, body weight of 45 to 80 kg for women and 60 to 90 kg for men. To address the impact of tobacco smoke exposure on urinary excretion of furan-dependent metabolites in humans, the study population included six non-smoking (3 males and 3 females) as well as four smoking subjects (2 males and 2 females).

#### Study design and sample collection

**Fig. 2:**
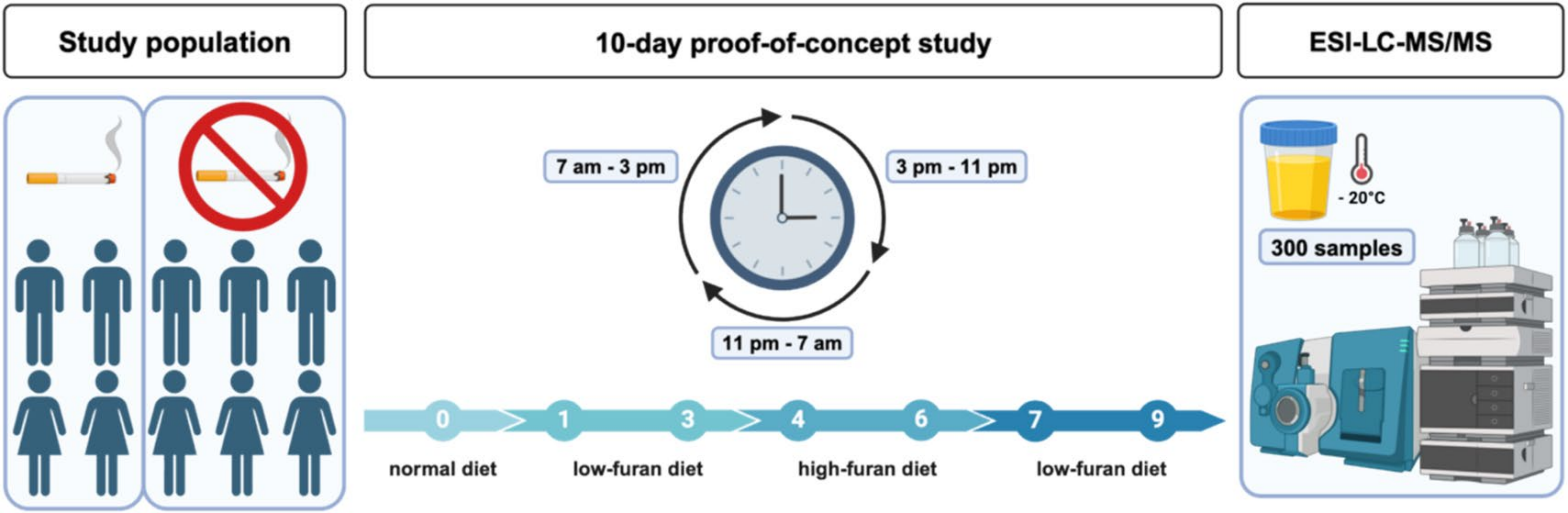
Study design of the 10-day human proof-of-concept study in ten healthy male (*n* = 5) and female (*n* = 5) volunteers. The study population included four smoking and six non-smoking study subjects. Urine samples were collected at 8 h intervals and analyzed by stable isotope dilution LC–MS/MS. (Created with Biorender.com)

The study lasted 10 days and was divided into three main segments. Participants consumed their regular diet for 24 h prior to the start of the study (day 0). During the first 3-day period, subjects followed a low-furan diet for three days (day 1–3). In the subsequent second part of the study, subjects were asked to predominantly consume food high in furan content for three days (day 4–6). In the final 3-day period, participants returned to a low-furan diet for a further three days (7–9) (Fig. 2). Dietary specifications and preparation methods for low-furan and high-furan study segments are detailed in chapter 2.2.3. Subjects maintained their food records during the entire study.

Urine was collected in 8 h intervals. On day 0, the morning urine was discarded before commencing the first collection period. Subjects fully emptied their bladder into a measuring cylinder and documented the volume. The urine was then transferred into a 2-L container and kept at 4 °C. Urine aliquots (2 × 80 mL) were taken at the end of each 8 h interval and stored at − 20 °C until further analysis.

#### Dietary specifications

Participants were provided with a list of food containing high and low furan levels and dietary recommendations for each study segment (Supplementary Table S1). During periods of low-furan diet, subjects had to strictly restrain from consuming coffee and coffee products. Low-furan diet exclusively consisted of fresh and unprocessed ingredients including fruit, vegetables, meat and fish prepared by steaming or boiling. Participants were also allowed to eat dairy products, rice and noodles. During periods of a high-furan diet, subjects were asked to consume predominantly canned or jarred foods. Meals were prepared by methods resulting in significant browning of the product such as baking, frying or toasting. Furthermore, subjects were requested to drink several cups of coffee per day. Participants documented all consumed beverages and foods in a nutrition diary.

#### Smoking

Smoking study subjects maintained their regular smoking habits throughout the entire study and reported the number of smoked cigarettes per day. The use of nicotine-replacement products was not permitted.

### Quantitative analysis of GSH-BDA

#### Sample preparation

Urine samples were thawed at 4–8 °C and diluted with water (LC–MS/MS grade) at a dilution factor of 10. To an aliquot of 90 µL diluted urine, 10 µL of internal standard solution and 1 µL of HCl (8 M) were added. The internal standard solution contained isotopically labeled [^13^C_2_^15^N_2_]-GSH-BDA (0.05 µM) dissolved in water. The samples were centrifuged at 4 °C and 15,000 g for 10 min.

#### LC–MS/MS analysis with column switching technique

The analytical LC–MS/MS method with column switching was adjusted based on previously described methods to ensure sensitive analysis of GSH-BDA in human urine (Kellert et al. 2008; Karlstetter and Mally 2020). The column-switching HPLC system comprised a trap column (Synergi Fusion-RP, 4 µm, 150 × 2 mm, 80 Å, Phenomenex Inc.), an analytical column (Synergi Polar-RP, 4 µm, 150 × 2 mm, 80 Å, Phenomenex Inc.) and two binary pumps, controlled by an electric ten-point switching valve. The mobile phase consisted of water (containing 0.1% (v/v) formic acid) as solvent A and methanol (containing 0.1% (v/v) formic acid) as solvent B. In the loading position, an injection volume of 85 µL was transferred onto the trap column by pump 1 at a constant flow rate of 1 mL/min and 100% A/0% B. While these conditions were maintained for 3.1 min on the trap column, the analytical column was equilibrated by pump 2 at 0.3 mL/min and 100% A/0% B. The electric valve subsequently switched to the elution position, allowing pump 2 to back-flush the analytes from the trap column onto the analytical column. Gradient elution started at 100% A/0% B and increased the content of solvent B to 20% A/80% B within 6.9 min. These conditions were held for 2 min, followed by a further increase of solvent B to 10% A/90% B. The gradient was returned to initial conditions of 100% A/0% B within 2 min and remained for a further 2 min. After a total run time of 11 min, the trap column was disconnected from the analytical column. The trap column was washed at a flow rate of 0.5 mL/min and 10% A/90% B for 6.5 min and was then immediately equilibrated at 100% A/0% B until the end of the run (22 min).

Mass spectrometric measurements were performed in positive electrospray ionization mode with an ion spray voltage of 5500 V and a source temperature of 500 °C. Nitrogen was used as ion spray (50 psi), drying gas (60 psi), curtain gas (40 psi) and collision gas. Analysis was carried out in multiple reaction monitoring (MRM) mode with a dwell time of 100 ms for each mass transition. Compound-specific ESI–MS/MS-parameters are given in Table 1. Analyst 1.7.3 software was used for data recording (Applied Biosystems/MDS Sciex, Darmstadt, Germany).

**Table 1:** Compound-specific LC–ESI–MS/MS parameters for furan-dependent metabolites, cotinine, caffeic acid and corresponding internal standards

Compound	RT (min)	Precursor ion (m/z)	Product ion (m/z)	DP (V)	EP (V)	CE (V)	CXP (V)
GSH-BDA	11.2	355.9	210.0 (Qn)	106	10	35	12
[^13^C_2_^15^N_1_]-GSH-BDA		359.2	210.2 (Qn)	91	10	33	14
			314.0 (Ql)		10	33	14
NAcLys-BDA	12.3	255.0	209.2 (Qn)	71	10	19	14
			167.2 (Ql)		10	25	20
[^13^C_6_^15^N_2_]-NAcLys-BDA		263.0	216.2 (Qn)	81	10	19	14
			174.1 (Ql)		10	25	20
NAcCys-BDA-NAcLys	12.6	400.1	271.1 (Qn)	96	10	23	16
			166.0 (Ql)		10	39	20
[^13^C_2_]-NAcCys-BDA-NAcLys		402.1	271.1 (Qn)	106	10	41	22
			166.0 (Ql)		10	35	16
NAcCys-BDA-NAcLys sulfoxide	11.5	416.1	269.0 (Qn)	106	10	19	16
			129.9 (Ql)		10	35	16
[^13^C_2_]-NAcCys-BDA-NAcLys sulfoxide		418.1	269.0 (Qn)	106	10	19	16
			132.0 (Ql)		10	35	16
Cotinine	2.1	177.1	80.0 (Qn)	90	10	33	10
			98.1 (Ql)		10	29	12
Cotinine-methyl-d_3_		180.0	80.1 (Qn)	111	10	35	12
			101.1 (Ql)		10	29	14
Caffeic acid	2.5	181.0	89.1 (Qn)	78	10	39	14
			117.0 (Ql)		10	29	14

Shown are retention time (RT), precursor ion, product ion (quantifier (Qn) and qualifier transition (Ql)), declustering potential (DP), entrance potential (EP), cell entrance potential (CEP), collision energy (CE) and cell exit potential (CXP)

As an analyte-free matrix was not available, the six-point calibration curve was constructed by adding appropriate volumes of two different working standard solutions to artificial urine prepared according to a previously published protocol (Sarigul et al. 2019). Working standard solutions containing 1 ng/mL and 10 ng/mL in water (LC–MS/MS grade) were obtained from a 100 ng/mL stock solution. Calibration was linear in the range of 0.1 to 3.2 ng/mL. To 90 µL of diluted urine, 10 µL internal standard solution and 1 µL HCl (8 M) were added. The solutions were mixed by vortexing and centrifuged at 4 °C and 15,000 g for 10 min. Quantitation was performed using the ratios of analyte peak area to internal standard peak area. Method precision was ensured by analyzing quality controls of 1.5 ng/mL every tenth sample. The limit of detection and quantitation were 0.02 ng/mL and 0.05 ng/mL, respectively.

### Quantitative analysis of NAcLys-BDA

#### Sample preparation

Aliquots of urine were thawed at 4–8 °C and vortexed. Urine was diluted with water at dilution factors of 2–5. A volume of 10 µL internal standard solution, containing NAc-[^13^C_6_^15^N_2_]-Lys-BDA (0.92 µM) in water, was added to 90 µL of diluted urine. The solution was acidified with 1 µL of 8 M HCl and centrifuged at 4 °C and 15,000 g for 10 min.

#### LC–MS/MS analysis

Quantitative analysis of NAcLys-BDA in human urine was performed according to a previously described method for rat urine analysis using a Triple Quad 5500 + QTRAP mass spectrometer (Applied Biosystems/MDS Sciex, Darmstadt, Germany) coupled to an Agilent 1100 HPLC and Agilent 1100 autosampler (Agilent, Waldbronn, Germany) (Kalisch et al. 2024). Chromatographic separation was carried out on a Synergi Polar-RP analytical column (4 µm, 150 × 2 mm, 80 Å, Phenomenex Inc.) with water containing 0.1% (v/v) formic acid) as solvent A and methanol (containing 0.1% (v/v) formic acid) as solvent B at a flow rate of 0.3 mL/min. Gradient elution started at 100% solvent A for 3 min, followed by a linear gradient to 20% A/80% within 7 min, which was then held for 2 min. The concentration of solvent A was further decreased to 10% A/90% B and held at 90% B for 2 min, before returning to the initial conditions of 100% A/0% B in 2 min. These conditions remained until the end of the run (22 min).

Mass spectrometric analysis was carried out using electrospray ionization in positive ion mode and MRM mode with an ion spray voltage of 5500 V and a source temperature of 500 °C. The dwell time was 170 ms for each mass transition. Nitrogen was used as ion spray (50 psi), drying gas (60 psi), curtain gas (40 psi) and collision gas. Compound-specific ESI–MS/MS-parameters are detailed in Table 1. Data were recorded using Analyst 1.7.3 software (Applied Biosystems/MDS Sciex, Darmstadt, Germany).

Preparation of the calibration curve was performed with minor modifications. Artificial urine was used as a matrix for the calibration standards (Sarigul et al. 2019). The seven-point calibration curve was prepared by spiking artificial urine with appropriate amounts of a 100 ng/mL working standard solution. Calibration was linear in the range of 1.25–80 ng/mL. To a volume of 90 µL of each calibration standard, 10 µL internal standard solution and 1 µL 8 M HCl were added. Calibration standards were then mixed and centrifuged at 4 °C and 15,000 g for 10 min. Method precision was confirmed by measuring a QC sample of 20 ng/mL after every tenth sample. The peak area ratios (analyte peak area/internal standard peak area) were used for quantitation. LOD and LOQ of NAcLys-BDA were determined in artificial urine as 0.49 ng/mL and 1.14 ng/mL, respectively.

### Quantitative analysis of NAcCys-BDA-Lys and NAcCys-BDA-Lys sulfoxide

#### Sample preparation

Urine sample preparation was performed using a modified version of a previously described method (Grill et al. 2015). Urine was thawed at 4–8 °C and thoroughly mixed. To a volume of 100 µL urine, 275 µL sodium bicarbonate solution (0.5 M) and 25 µL acetic anhydride were added. Samples were mixed by vortexing and were immediately uncapped before incubation for 45 min at RT. Samples were diluted with water (LC–MS/MS grade) at dilution factors of 8–40 to achieve concentrations within the linear range of the calibration curve for both analytes. An aliquot of 90 µL acetylated and diluted urine was fortified with 10 µL internal standard mix, containing [^13^C_2_]-NAcCys-BDA-NAcLys (0.04 µM) and [^13^C_2_]-NAcCys-BDA-NAcLys sulfoxide (0.04 µM). Solutions were centrifuged at 4 °C and 15,000 g for 10 min.

#### LC–MS/MS analysis with column switching technique

LC–MS/MS analysis was performed with a Triple Quad 5500 + QTRAP mass spectrometer (Applied Biosystems/MDS Sciex, Darmstadt, Germany) coupled to an Agilent 1100 HPLC and Agilent 1100 autosampler (Agilent, Waldbronn, Germany). Liquid chromatography was performed using the same gradient elution and column switching technique as described for GSH-BDA (2.3.2).

Mass spectrometric analysis was conducted using electrospray ionization operating in positive ion mode and multiple reaction monitoring (MRM) mode with an ion spray voltage of 5500 V and a source temperature of 500 °C. Nitrogen was used as ion spray (50 psi), drying gas (60 psi), curtain gas (40 psi) and collision gas. Compound-specific ESI–MS/MS-parameters are given in Table 1. Dwell time was set at 80 ms for each mass transition. Analyst 1.7.3 software was used for data recording (Applied Biosystems/MDS Sciex, Darmstadt, Germany).

As NAcCys-BDA-Lys and NAcCys-BDA-Lys sulfoxide were indirectly measured as their *N*-acetylated species, NAcCys-BDA-NAcLys and NAcCys-BDA-NAcLys sulfoxide were used as reference standards for quantification. Seven-point calibration curves were prepared by spiking artificial urine with appropriate amounts of working standard solution, containing 10 ng/mL of each metabolite (Sarigul et al. 2019). Calibration was linear in the range of 0.0735–4.71 ng/mL. To 90 µL of each calibration standard, 10 µL of internal standard mix was added. Samples were centrifuged at 15,000 g and 4 °C for 10 min. Quantitation was conducted using peak area ratios (analyte peak area/internal standard peak area). To ensure precision during the analytical run, quality controls, containing 2.35 ng/mL of each analyte, were measured for every tenth sample. LOQs for NAcCys-BDA-NAcLys and NAcCys-BDA-NAcLys sulfoxide were determined in artificial urine as 0.06 and 0.07 ng/mL, respectively. The LOD was defined as 0.03 ng/mL for both metabolites.

### Quantitative analysis of cotinine

#### Sample preparation

Urine samples were thawed at 4–8 °C and thoroughly mixed. Urine was diluted with water (LC–MS/MS grade) to achieve a 1:10 dilution. An aliquot of 200 µL diluted urine was fortified with 20 µL internal standard solution, containing 250 ng/mL cotinine-methyl-d_3_ in methanol. Samples were centrifuged for 10 min at 4 °C and 15,000 g.

#### LC–MS/MS analysis

LC–MS/MS analysis was performed with as Triple Quad 5500 + QTRAP mass spectrometer (Applied Biosystems/MDS Sciex, Darmstadt, Germany) coupled to an Agilent 1100 HPLC and Agilent 1100 autosampler (Agilent, Waldbronn, Germany). Chromatographic separation was carried out on a Synergi Polar-RP analytical column (4 µm, 150 × 2 mm, 80 Å, Phenomenex Inc.). The injection volume was 10 µL per sample. The mobile phase was a gradient of water (containing 0.1% (v/v) formic acid) as solvent A and methanol (containing 0.1% (v/v) formic acid) as solvent B at a flow rate of 0.3 mL/min. Solvent A was maintained at 90% for 2 min, followed by a linear gradient to 60% A/40% B within 3 min. The gradient was then returned to initial conditions of 90% A/10% B within 1 min and held for another 2 min until the end of the run (8 min).

Mass spectrometric analysis was performed using electrospray ionization operating in positive ion mode and multiple reaction monitoring (MRM) mode with an ion spray voltage of 5500 V and a source temperature of 500 °C. Nitrogen was used as ion spray (50 psi), drying gas (60 psi), curtain gas (40 psi) and collision gas. Compound-specific ESI–MS/MS-parameters are given in Table 1. Dwell time was 250 ms for each mass transition. Data were recorded by Analyst 1.7.3 software (Applied Biosystems/MDS Sciex, Darmstadt, Germany).

Seven-point calibration curves were prepared by spiking analyte-free matrix with appropriate amounts of working standard solution, containing 100 ng/mL cotinine in water (LC–MS/MS grade). Calibration was linear in the range of 1.5 to 80 ng/mL. Analyte free matrix was obtained by pooling the urine of three male and female non-smoking subjects. The pooled urine was screened for endogenous interferences and diluted with water at a dilution factor of 10. Calibration standards (200 µL) were fortified with 20 µL internal standard solution, vortexed and centrifuged at 4 °C and 15,000 g for 10 min. The peak area ratios (analyte peak area/internal standard peak area) were used for quantitation. LOD and LOQ were determined as 1 ng/mL and 3 ng/mL, respectively.

### Quantitative analysis of caffeic acid

#### Sample preparation

Urine samples were thawed at 4–8 °C and vortexed. Enzymatic hydrolysis and liquid–liquid extraction were performed as previously described (Ito et al. 2005). A volume of 250 µL was acidified with 20 µL acetic acid (0.58 M) and treated with 15.3 µL of β-glucuronidase type H-2 from *H. pomatia* (≥ 85,000 units/mL) in water. The mixture was incubated at 37 °C for 1 h with constant shaking before enzymatic hydrolysis was stopped by adding 1 µL HCl (10 M). Urine samples were then extracted with 700 µL ethyl acetate by vortexing. After centrifugation at 5,000 g for 10 min, an aliquot of 570 µL of the organic layer was evaporated to dryness and reconstituted in 200 µL methanol–water (95:5).

#### LC–MS/MS analysis

LC–MS/MS analysis was performed using a Triple Quad 5500 + QTRAP mass spectrometer (Applied Biosystems/MDS Sciex, Darmstadt, Germany) connected to an Agilent 1100 HPLC and Agilent 1100 autosampler (Agilent, Waldbronn, Germany). Chromatographic separation was carried out on a Synergi Luna Omega PS C18 analytical column (3 µm, 150 × 2.1 mm, 100 Å, Phenomenex Inc.) with water (containing 0.1% (v/v) formic acid) as solvent A and methanol (containing 0.1% (v/v) formic acid) as solvent B at a constant flow rate of 0.25 mL/min. Gradient elution started with 5% A/95% B for 2 min, followed by a linear gradient to 95% A/5% B in 4 min. These conditions were held for 2 min before decreasing to 5% A/95% B within 2 min. The column was re-equilibrated at initial conditions for an additional 2 min. The total chromatographic run time was 12 min.

Mass spectrometric analysis was carried out using electrospray ionization operating in positive ion mode and multiple reaction monitoring (MRM) mode with an ion spray voltage of 5500 V and a source temperature of 500 °C. Nitrogen was used as ion spray (50 psi), drying gas (60 psi), curtain gas (40 psi) and collision gas. Compound-specific ESI–MS/MS-parameters are given in Table 1. Dwell time was 180 ms for each mass transition. Data processing was performed with Analyst 1.7.3 software (Applied Biosystems/MDS Sciex, Darmstadt, Germany).

The calibration curve was constructed at six concentration levels (10 – 160 ng/mL) using two different working solutions of 1600 ng/mL and 400 ng/mL caffeic acid in methanol. Calibration standards were prepared by adding appropriate amounts of the respective working solution to a methanol–water mixture (95:5). Solutions were centrifuged at 5,000 g and 4 °C for 10 min.

### Calculation of probable daily furan intakes based on urinary metabolite excretion

Probable daily furan intakes (PDI) were calculated based on urinary excretion of the furan-dependent metabolites GSH-BDA, NAcLys-BDA, NAcCys-BDA-Lys and NAcCys-BDA-Lys sulfoxide in smoking and non-smoking subjects using relative excretion rates of the respective metabolites previously determined in male and female F344/DuCrl rats (Kalisch et al. 2024). To estimate daily furan intakes based on human NAcCys-BDA-Lys and NAcCys-BDA-Lys sulfoxide excretion, relative excretion rates of the corresponding *N*-acetylated metabolites NAcCys-BDA-NAcLys and NAcCys-BDA-NAcLys sulfoxide were used, as rats primarily excrete the *N*-acetylated species. Consequently, calculated daily furan intakes were normalized to the subjects’ individual body weight. PDIs were calculated according to the following equation, where A is the absolute urinary metabolite excretion in µg per day, W is the individual body weight in kg, M is the molar mass in g/mol and E is the relative excretion rate of the respective metabolite in % of the externally applied furan dose. Relative excretion rates of the metabolites established in male and female F344/DuCrl rats (Kalisch et al. 2024) are shown in Table 2. Mean GSH-BDA excretion rates in humans (male: 2.85%; female: 3.10%) were taken from the literature (Bohlen et al. 2024).

$${\text{PDI}} \left( {{\mu g}/{\text{kg bw}}/{\text{d}}} \right) = \frac{{\text{A}}}{{{\text{E}} \times {\text{W}}}} \times \frac{{{\text{M}} ({\text{Furan}})}}{{{\text{M}} ({\text{Metabolite}})}}$$

**Table 2:** Mean relative 24h-excretion rates of the furan-dependent metabolites GSH-BDA, NAcLys-BDA, NAcCys-BDA-NAcLys and NAcCys-BDA-NAcLys sulfoxide determined in male and female F344/DuCrl rats (Kalisch et al. 2024)

Metabolite	Mean relative 24h-excretion rate in male rats	Mean relative 24h-excretion rate in female rats
*GSH-BDA*	2.18%^1^	1.18%^1^
*NAcLys-BDA*	0.59%^2^	0.52%^2^
*NAcCys-BDA-NAcLys*	1.58%^1^	2.14%^1^
*NAcCys-BDA-NAcLys sulfoxide*	0.69%^1^	0.28%^3^

^1^Mean relative 24h-excretion rate determined dose groups 1 – 1000 µg/kg bw

^2^Relative 24h-excretion rate determined in highest dose group 1000 µg/kg bw

^3^Mean relative 24h-excretion rate determined in dose groups 10 – 1000 µg/kg bw

A, Absolute metabolite excretion (µg/d), E, Relative excretion rate (%), W, Individual body weight (kg), M, Molar mass (g/mol)

## Results

**Fig. 3:**
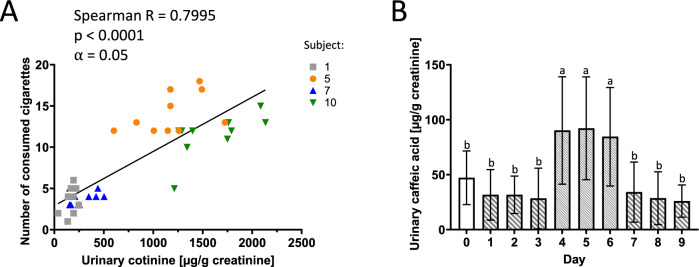
(A) Correlation between urinary 24 h cotinine excretion and the number of consumed cigarettes in smoking subjects (*n* = 4). Correlation analysis was performed using Spearman’s rank correlation coefficient R (*⍺* = 0.05; *p* < 0.05). (B) Mean urinary 24 h caffeic acid excretion in ten healthy male (*n* = 5) and female (*n* = 5) subjects, demonstrating a clear increase in response to coffee consumption during the high-furan diet interval on days 4–6) Data are presented as mean ± SD. Statistical comparison using repeated measure one-way ANOVA with Tukey test (post-hoc) at a significance level of *⍺* = 0.05. Different letters (a-b) indicate significant differences (*p* < 0.05)

To assess the contribution of coffee and cigarette consumption as major dietary and non-dietary sources of furan exposure on furan metabolite excretion, caffeic acid and cotinine were quantified in the urine of human volunteers as biomarkers of coffee and cigarette consumption. As expected, cotinine was not detected in the urine of non-smoking individuals. A strong positive correlation between concentrations of cotinine in the urine of active smokers and the self-reported number of consumed cigarettes was observed (Fig. 3 A). Although caffeic acid was also found in urine during periods of low-furan diet, presumably due to dietary intake of caffeic acid from other polyphenol-rich foods, urinary caffeic acid excretion showed a clear increase in response to coffee consumption during the high-furan diet interval (Fig. 3 B). These findings support the use of both cotinine and caffeic acid as reasonable markers of cigarette and coffee consumption.

A stable isotope dilution ESI-LC–MS/MS method previously developed for quantitative analysis of furan metabolites in rat urine (Kalisch et al. 2024) was adapted and optimized for each analyte separately to ensure accurate quantification of furan-derived metabolites in the urine of human volunteers exposed to furan via food. To further enhance the sensitivity, a column-switching unit was integrated into the analytical LC–MS/MS set-up for quantitative analysis of GSH-BDA in human urine. In contrast to rats, the cysteine-BDA-lysine crosslinks excreted in human urine are *N*-acetylated only at the cysteine residue, i.e. as NAcCys-BDA-Lys and NAcCys-BDA-Lys sulfoxide, whereas NAcCys-BDA-NAcLys and NAcCys-BDA-NAcLys sulfoxide were not detected. Due to interfering contaminants in human urine, quantitative analysis of NAcCys-BDA-Lys and NAcCys-BDA-Lys sulfoxide required conversion to their completely *N*-acetylated form, i.e. NAcCys-BDA-NAcLys and NAcCys-BDA-NAcLys sulfoxide, via acetic anhydride as previously described (Grill et al. 2015) and integration of a column-switching unit to further improve the sensitivity.

**Fig. 4:**
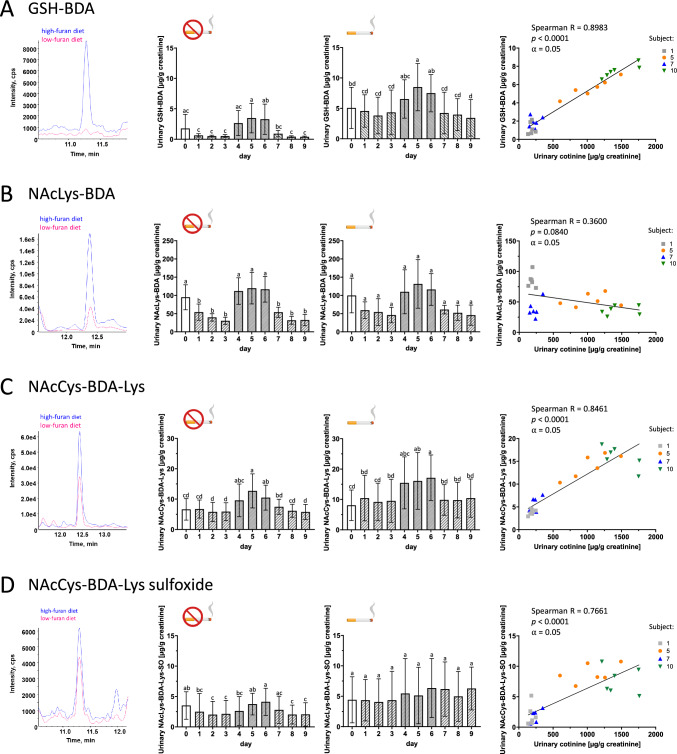
Quantitative analysis of GSH-BDA (**A**), NAcLys-BDA (**B**), NAcCys-BDA-Lys as NAcCys-BDA-NAcLys (**C**) and NAcCys-BDA-Lys sulfoxide as NAcCys-BDA-NAcLys sulfoxide (**D**) in human urine. The figure includes representative chromatograms obtained from a non-smoking female subject during high- and low-furan diet, urinary 24 h metabolite excretion of non-smoking and smoking subjects (mean ± SD), and the correlation between urinary 24 h metabolite excretion during low-furan diet and 24 h cotinine excretion in smoking subjects. Statistical comparison of excretion data was performed using repeated measure one-way ANOVA with Tukey test (post-hoc) at a significance level of *⍺* = 0.05. Different letters (a–d) indicate significant differences (*p* < 0.05). Correlation was analyzed using Spearman’s rank correlation coefficient R (*⍺* = 0.05; *p* < 0.05). Subject 2 was excluded from the analysis of GSH-BDA

LC–MS/MS analysis confirmed the presence of GSH-BDA, NAcLys-BDA, as well as NAcCys-BDA-Lys and its corresponding sulfoxide in human urine (Fig. 4), whereas the fully *N-*acetylated BDA-derived cysteine-lysine crosslinks NAcCys-BDA-NAcLys and NAcCys-BDA-NAcLys sulfoxide were consistently below the limits of detection in urine of human volunteers. NAcLys-BDA was identified as the most abundant furan metabolite in human urine, with more than one order of magnitude higher baseline concentrations at the onset of the study (day 0) compared to the other metabolites (Fig. 4B).

**Fig. 5:**
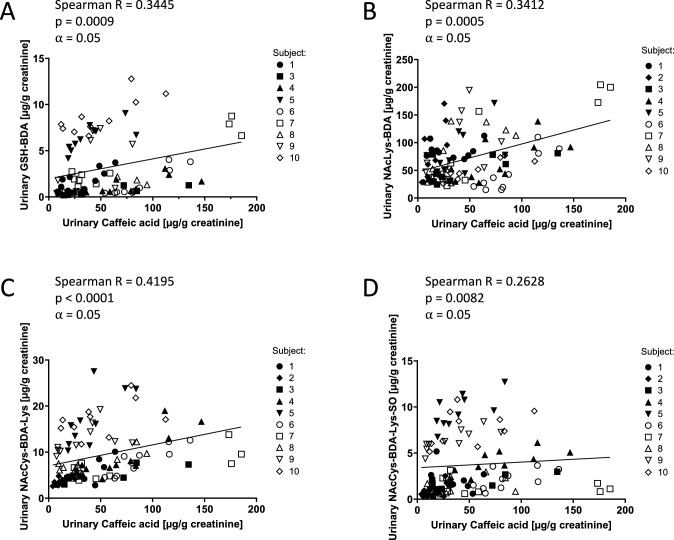
Correlation between urinary 24 h metabolite excretion and urinary 24 h caffeic acid excretion: GSH-BDA (**A**), NAcLys-BDA (**B**), NAcCys-BDA-Lys (**C**) and NAcCys-BDA-Lys sulfoxide (**D**). Correlation was analyzed using Spearman’s rank correlation coefficient R (*⍺* = 0.05; *p* < 0.05). Correlation analysis includes data points obtained during a normal, low-furan and high-furan diet. Subject 2 was excluded from the correlation analysis of GSH-BDA. Filled symbols represent male study participants (1–5), open symbols represent female participants (6–10)

A significant increase in urinary excretion of GSH-BDA, which was identified as the most promising biomarker in our previous study in rats (Kalisch et al. 2024), was observed in response to dietary furan intake irrespective of the subjects´ smoking status (Fig. 4 A). In line with increased excretion of GSH-BDA in response to a high-furan diet, a weak but significant correlation between GSH-BDA excretion and coffee consumption, reflected by analysis of urinary caffeic acid, was demonstrated in both smoking and non-smoking subjects (Spearman R = 0.3445) (Fig. 5A). Urinary GSH-BDA concentrations in one individual (Subject 2, male, non-smoking) were approximately ten-fold higher compared to those of the remaining subjects. A source for these high levels of GSH-BDA could not be identified, and the individual was excluded from further analysis.

During the low furan diet, GSH-BDA levels were below the limit of detection in the urine of non-smokers. Based on the LOD of 0.02 ng/mL, non-smoking subjects were calculated to excrete GSH-BDA at ≤ 0.56 ± 0.31 µg/g creatinine per day while on a low furan diet. Urinary GSH-BDA excretion significantly increased about 5.6-fold to 3.14 ± 2.19 µg/g creatinine during periods of a high-furan diet, and rapidly declined upon returning to a low-furan diet (Fig. 4A, Table 3).

**Table 3:** Urinary 24 h metabolite excretion of non-smoking (*n* = 6) and smoking subjects (*n* = 4) as nmol and µg normalized to urinary creatinine

Metabolite	Diet type		Non-smoking subjects		Smoking subjects	
			mean (± SD)	min.–max	mean (± SD)	min. – max
GSH-BDA	normal	nmol/g creatinine	4.98 ± 6.47	0.51–16.1	14.3 ± 9.5	4.9–23.1
		µg/g creatinine	1.76 ± 2.30	0.18–5.71	5.06 ± 3.38	1.74–8.19
	low-furan	nmol/g creatinine	1.59 ± 0.87	0.53–5.06	11.4 ± 7.8	1.6–24.4
		µg/g creatinine	0.56 ± 0.31	0.19–1.80	4.03 ± 2.73	0.58–8.59
	high-furan	nmol/g creatinine	8.83 ± 6.16	1.84–20.9	21.1 ± 8.9	7.1–36.0
		µg/g creatinine	3.14 ± 2.19	0.65–7.42	7.48 ± 3.15	2.53–12.71
NAcLys-BDA	normal	nmol/g creatinine	373.6 ± 135.3	144.6–546.6	392.2 ± 187.6	203.3–615.9
		µg/g creatinine	94.9 ± 34.4	36.7–138.8	99.6 ± 47.7	51.6–156.4
	low-furan	nmol/g creatinine	158.8 ± 66.0	58.3–337.3	209.0 ± 89.4	87.1–421.8
		µg/g creatinine	40.3 ± 16.8	14.8–85.7	53.1 ± 22.7	22.1–107.1
	high-furan	nmol/g creatinine	457.0 ± 142.2	241.4–766.4	469.2 ± 208.5	261.2–805.0
		µg/g creatinine	116.1 ± 36.1	61.3–194.7	119.2 ± 53.0	66.3–204.5
NAcCys-BDA-Lys	normal	nmol/g creatinine	18.6 ± 9.89	8.11–33.7	22.6 ± 14.1	7.91–36.9
		µg/g creatinine	6.62 ± 3.53	2.90–12.02	8.07 ± 5.02	2.82–13.18
	low-furan	nmol/g creatinine	17.6 ± 7.27	7.33–34.5	27.6 ± 15.9	8.15–52.5
		µg/g creatinine	6.29 ± 2.59	2.62–12.32	9.86 ± 5.66	2.91–18.74
	high-furan	nmol/g creatinine	30.8 ± 13.5	11.7–53.9	45.4 ± 21.6	18.9–77.1
		µg/g creatinine	10.93 ± 5.0	2.79–19.26	16.22 ± 7.73	6.73–27.53
NAcCys-BDA-Lys sulfoxide	normal	nmol/g creatinine	8.44 ± 5.45	2.26–17.5	10.6 ± 9.12	1.91–22.1
		µg/g creatinine	3.51 ± 2.26	0.94–7.26	4.42 ± 3.78	0.79–9.16
	low-furan	nmol/g creatinine	5.43 ± 5.02	0.63–20.3	12.1 ± 8.87	1.36–26.0
		µg/g creatinine	2.25 ± 2.08	0.26–8.41	5.03 ± 3.68	0.56–10.79
	high-furan	nmol/g creatinine	8.44 ± 5.06	0.77–18.1	13.6 ± 11.2	1.44–30.7
		µg/g creatinine	3.49 ± 2.11	0.32–7.52	5.64 ± 4.65	0.60–12.72

Data are presented as mean (± SD) and range (min. – max.). Subject 2 (male, non-smoker) was excluded in GSH-BDA excretion data

In contrast to non-smoking subjects, GSH-BDA was detectable in urine samples of smoking subjects throughout the entire study. During periods of a low-furan diet, urinary GSH-BDA excretion of smokers accounted for 4.03 ± 2.73 µg/g creatinine per day and increased by about twofold to a mean daily excretion of 7.48 ± 3.15 µg/g creatinine when subjects adhered to a high-furan diet (Table 3). Importantly, GSH-BDA excretion was significantly higher in smoking subjects compared to non-smoking subjects, indicating cigarette smoking as a non-dietary source for this metabolite (Fig. 4A). This was further confirmed by a strong positive correlation between urinary GSH-BDA and tobacco smoke exposure determined by the analysis of urinary cotinine (Spearman *R* = 0.8983) (Fig. 4A).

In contrast to GSH-BDA, urinary excretion of NAcLys-BDA was similar in smoking and non-smoking subjects, suggesting that this biomarker may not be significantly affected by smoking (Fig. 4B). This was further supported by the lack of correlation between urinary NAcLys-BDA and urinary cotinine levels in smoking subjects (Fig. 4B). During periods of low dietary furan intake, mean urinary excretion of NAcLys-BDA was 40.3 ± 16.8 µg/g creatinine in non-smokers and 53.1 ± 22.7 µg/g creatinine in smokers (Fig. 4B, Table 3). A notable, 2–threefold increase in urinary NAcLys-BDA excretion was observed in non-smokers and smokers after consumption of a high-furan diet, followed by a gradual decrease when subjects returned to a low-furan diet. Following a high-furan diet regime, non-smoking subjects excreted on average 116.1 ± 36.1 µg NAcLys-BDA /g creatinine via urine, while urinary excretion of smokers was 119.2 ± 53.0 µg/g creatinine (Fig. 4B, Table 3). In line with the observed increase in NAcLys-BDA excretion following consumption of a diet high in furan content, a weak correlation between urinary NAcLys-BDA and caffeic acid as a marker for coffee consumption as a main contributor to furan exposure was demonstrated in both non-smoking and smoking subjects (Spearman *R* = 0.3412) (Fig. 5B).

A slight but significant increase in NAcCys-BDA-Lys excretion was also observed in non-smoking and smoking subjects in response to increased dietary furan intake (Fig. 4C). The impact of coffee consumption during the high-furan diet interval on urinary excretion of NAcCys-BDA-Lys was further underscored by a moderate correlation between NAcCys-BDA-Lys levels and urinary caffeic acid (Spearman R = 0.4195) (Fig. 5C). During periods of low-furan diet, NAcCys-BDA-Lys was excreted at 6.29 ± 2.59 µg/g creatinine per day in non-smoking subjects and increased to 10.93 ± 5.0 µg/g creatinine when the diet regime was changed to a high-furan diet (Fig. 4C, Table 3). Similar to GSH-BDA, excretion of NAcCys-BDA-Lys was higher in smokers relative to non-smokers. Smoking subjects exhibited a mean daily NAcCys-BDA-Lys excretion of 9.86 ± 5.66 µg/g creatinine during periods of low-furan diet, which increased to 16.22 ± 7.73 µg/g creatinine per day following consumption of a high-furan diet (Fig. 4C, Table 3). Consistent with these findings, a strong positive correlation was observed between urinary NAcCys-BDA-Lys and smoking as determined by analysis of urinary cotinine (Spearman R = 0.8461) (Fig. 4C), suggesting cigarette smoking as a non-dietary source of exposure.

LC–MS/MS analysis of NAcCys-BDA-Lys sulfoxide in human urine revealed no significant impact of dietary furan intake on NAcCys-BDA-Lys sulfoxide excretion in smokers, while minor effects of furan intake were observed in non-smokers. This was also evident from the weak correlation between urinary excretion of NAcCys-BDA-Lys sulfoxide and coffee consumption (Spearman R = 0.2628) (Fig. 4D and Fig. 5D). However, urinary excretion of NAcCys-BDA-Lys sulfoxide strongly correlated with tobacco smoke exposure as determined by analysis of urinary cotinine (Spearman R = 0.7661) (Fig. 4D). Levels of NAcCys-BDA-Lys sulfoxide measured during periods of low-furan diet were about twofold higher in the urine of smokers than in non-smokers, with mean daily excretions of 5.03 ± 3.68 µg/g creatinine in smokers and 2.25 ± 2.08 µg/g creatinine in non-smoking study participants (Table 3). Of note, this increase in mean daily excretion of NAcCys-BDA-Lys sulfoxide in smokers compared to non-smokers was driven entirely by smokers that consumed > 10 cigarettes per day, whereas urinary concentrations of NAcCys-BDA-Lys sulfoxide in smokers that consumed < 10 cigarettes per day were within the range of non-smoking subjects.

Excretion of NAcCys-BDA-Lys sulfoxide in non-smokers slightly increased to 3.49 ± 2.11 µg/g creatinine following a high-furan diet, whereas excretion of NAcCys-BDA-Lys sulfoxide in smokers during periods of high-furan diet was similar to that during the low-furan diet, accounting for 5.64 ± 4.65 µg/g creatinine per day (Fig. 4D, Table 3).

To estimate the daily human exposure to furan from food, relative excretion rates of the respective metabolites previously determined in male and female F344/DuCrl rats (Kalisch et al. 2024), were applied to biomarker concentrations in human urine measured in the present study to calculate probable daily intakes (PDI).

Based on the relative GSH-BDA excretion rate of 2.18% of the external dose determined in male rats, calculated PDIs of non-smoking subjects ranged from 0.05 to 0.31 µg/kg bw/d during periods of low-furan diet and increased to 0.18–1.20 µg/kg bw/d when subjects increased their dietary furan intake via consumption of a high-furan diet (Table 4; Supplementary Tables S3 – S6). At the start of the study, when study participants adhered to their usual diet habits, furan exposure of non-smokers was estimated at 0.05–0.90 µg/kg bw/d (Table 4; Supplementary Tables S3 – S6). These PDIs calculated based on human urinary GSH-BDA excretion corresponded very well with exposure estimates in adults based on data from European dietary surveys, which range between 0.11 to 0.75 µg/kg bw per day (minimum LB to maximum UB) for average and 0.20 to 1.22 µg/kg bw per day (minimum LB to maximum UB) for highly exposed consumers (95th percentile), with coffee consumption as the main contributor to furan exposure in this age group (EFSA 2017).

**Table 4:** Probable daily furan intakes (µg/kg bw/d) of non-smoking (*n* = 5) and smoking subjects (*n* = 4) based on urinary GSH-BDA excretion and relative excretion rates of GSH-BDA previously determined in male and female F344/DuCrl rats (Kalisch et al. 2024), as well as excretion rates determined in humans (Bohlen et al. 2024)

Diet type	Probable daily furan intakes (µg/kg bw/d) Calculated based on relative excretion rates in							
	Male rats^1^		Female rats^2^		Male humans^3^		Female humans^4^	
	Mean ± SD	Min.–Max	Mean ± SD	Min.–Max	Mean ± SD	Min.–Max	Mean ± SD	Min.–Max
*Non-smoking subjects*								
Normal	0.33 ± 0.35	0.05–0.90	0.60 ± 0.65	0.09–1.67	0.25 ± 0.27	0.04–0.69	0.23 ± 0.25	0.04–0.64
Low-furan	0.12 ± 0.06	0.05–0.31	0.22 ± 0.10	0.09–0.57	0.09 ± 0.04	0.04–0.24	0.08 ± 0.04	0.03–0.22
High-furan	0.63 ± 0.35	0.18–1.20	1.16 ± 0.65	0.32–2.22	0.48 ± 0.27	0.13–0.92	0.44 ± 0.25	0.12–0.84
*Smoking subjects*								
Normal	1.12 ± 0.74	0.47–2.00	2.07 ± 1.36	0.88–2.71	0.86 ± 0.56	0.36–1.53	0.79 ± 0.52	0.39–1.40
Low-furan	0.81 ± 0.54	0.13–1.67	1.49 ± 0.99	0.23–3.09	0.62 ± 0.41	0.10–1.28	0.57 ± 0.38	0.09–1.18
High-furan	1.64 ± 0.71	0.52–2.70	3.03 ± 1.32	0.97–4.98	1.25 ± 0.55	0.40–2.06	1.15 ± 0.50	0.37–1.90

Data are presented as mean ± SD and range (min.–max.) after consumption of regular diet and diets with low and high furan content. Subject 2 (male, non-smoker) was excluded

^1^Relative excretion rate determined in male F344/DuCrl: 2.18%

^2^Relative excretion rate determined in female F344/DuCrl: 1.18%

^3^Relative excretion rate determined in male humans published by Bohlen et al. (2024). PDIs were calculated based on an excretion rate of 2.85% (average of excretion rates determined on days 4 and 8)

^4^Relative excretion rate determined in female humans published by Bohlen et al. (2024). PDIs were calculated based on an excretion rate of 3.10% (average of excretion rates determined on days 4 and 8)

Due to the higher urinary excretion of GSH-BDA in smoking subjects, calculated PDIs of smokers were significantly higher than those of non-smokers. Based on the relative GSH-BDA excretion rate of male rats (2.18% of the external dose), PDIs of smokers ranged from 0.13 to 2.70 µg/kg bw/d, thus exceeding the maximum 95th exposure estimate of 1.22 µg/kg bw/d in adults, as well as the maximum 95th exposure estimate of 1.82 µg/kg bw/d in infants (EFSA 2017) (Table 4). While these data indicate a significant contribution of smoking to overall furan exposure, the calculated PDIs should be interpreted with caution. Due to the limited information on GSH-BDA excretion following furan exposure via inhalation, the use of relative excretion rates determined in rats after oral application may not be suitable to estimate furan exposure from cigarette smoke. Calculation of PDIs based on the lower GSH-BDA excretion rate of 1.18% of the external dose in female rats led to approximately twofold higher exposure estimates, ranging from 0.09 to 2.22 µg/kg bw/d for non-smokers and 0.23–4.98 µg/kg bw/d for smokers (Table 4). Slightly lower PDIs were derived based on human excretion rates reported by Bohlen et al. (2024) (Table 4).

In contrast to GSH-BDA, calculation of PDIs based on human NAcLys-BDA excretion and relative NAcLys-BDA excretion rates in male (0.59% of the external dose) and female (0.52% of the external dose) rats (Kalisch et al. 2024) led to unrealistic furan exposure estimates (up to 231.5 µg/kg bw/d in non-smokers and 299 µg/kg bw/d in smokers) (Supplementary Table S2). Considering the low excretion rates of NAcLys-[^13^C_4_]-BDA in rats following a single dose of [^13^C_4_]-furan vs. the high background levels of NAcLys-BDA in urine of control rats, which resemble the high abundance of this metabolite in humans, we consider that NAcLys-BDA in urine unlikely derives from furan intake via food alone and suspect a yet unidentified exogenous or endogenous source for this metabolite. A dietary source may be supported by the increase in NAcLys-BDA excretion in response to a furan-rich diet.

Similarly, the estimation of furan exposure based on the urinary excretion of BDA-derived cysteine-lysine crosslinks was highly impacted by smoking and resulted in PDIs that significantly exceeded human exposure estimates published by EFSA (2017) (Supplementary Table S2). In light of the observed background of these metabolites in rats, which may also occur in humans, and the fact that our calculations were based on relative excretion rates of the completely *N*-acetylated metabolites in rats, we consider that analysis of NAcCys-BDA-Lys and NAcCys-BDA-Lys in human urine is unlikely to provide accurate estimates of human exposure to furan.

## Discussion

This human pilot study was designed to test if analysis of furan-dependent metabolites in human urine presents a suitable approach to monitor human exposure to furan and to investigate which biomarker(s) may provide reliable estimates of furan intake via food. Via administration of isotopically labeled [^13^C_4_]-furan to rats and simultaneous analysis of unlabeled and [^13^C_4_]-furan dependent metabolites in rat urine, we previously demonstrated significant background excretion of NAcLys-BDA and BDA-derived cysteine-lysine crosslinks in urine of control animals and considered that these metabolites are, therefore, unlikely to present suitable biomarkers of furan exposure (Kalisch et al. 2024). Although excretion of NAcLys-BDA and NAcCys-BDA-Lys in our human pilot study increased in response to high furan intake, exposure estimates based on the abundance of these metabolites in human urine and relative excretion rates determined in F344/DuCrl rats by far exceeded current human exposures reported by EFSA (2017). In contrast, quantitative analysis of GSH-BDA, which exhibited no background in rats and increased in human urine during periods of high furan intake, resulted in probable daily intakes (PDIs) in the range of exposure estimates derived from furan content in food and consumption data. These data suggest that monitoring GSH-BDA in human urine may provide an alternative or complementary approach to exposure assessment of furan in food, but also highlight furan exposure due to cigarette smoking as a confounding factor.

Our findings on GSH-BDA and its suitability as a biomarker of furan exposure are in line with recent investigations of furan metabolites in human urine following furan exposure via coffee. In the study by Kremer et al. (2023), ten healthy, nonsmoking volunteers received standardized meals prepared to avoid furan contamination for four days and then consumed a defined volume of coffee brew with a known furan content of 0.648 µmol/500 ml (Kremer et al. 2023). Urinary excretion of GSH-BDA clearly increased in response to coffee intake, accounting for 0.003 µmol within 24 h (< 1% of the ingested dose). The 24 h excretion rate of GSH-BDA reported by Kremer et al. (2023) is thus in the range of GSH-BDA excretion rates in our present study, accounting for an average of 0.003 µmol/24 h in non-smokers consuming a low-furan diet and increasing to 0.014 µmol/24 h following consumption of a furan-rich diet. In a duplicate diet study conducted by the same group (Bohlen et al. 2024), study participants were maintained on a low-furan diet for eleven days (controlled by furan analysis of meals prepared in duplicate) but received a defined amount of furan via coffee brew on days 4 (17.2 μg = 253 nmol) and 8 (46 μg = 676 nmol). Urinary excretion of GSH-BDA rapidly increased in a dose-related manner after furan intake via coffee consumption (t_max_: 3 h) and declined to pre-exposure levels within 12 h. Depending on gender and study day, excretion of GSH-BDA accounted for 2.4–3.7% of the administered dose (Bohlen et al. 2024). These excretion rates in humans are consistent with the low excretion rates of GSH-BDA in rats treated with [^13^C_4_]-furan (♀ 1.18% and ♂ 2.18% of the external dose) (Kalisch et al. 2024). Based on urinary GSH-BDA excretion in the present study and relative excretion rates (2.85% in male and 3.10% in female volunteers) taken from Bohlen et al. (2024), PDIs for non-smokers were estimated to range from 0.08 to 0.09 µg/kg bw/d during low furan diet and from 0.44 to 0.48 µg/kg bw/d during a high furan diet. For smoking subjects, PDIs calculated based on human excretion rats reported by Bohlen et al. (2024) were 0.57–0.62 µg/kg bw/d during a low-furan diet, and increased to 1.15–1.25 µg/kg bw/d following consumption of a high furan diet (Table 4). The slightly higher excretion rates in humans based on the study by Bohlen et al. (2024) result in slightly lower PDIs compared to those calculated using relative excretion rates determined in rats. In contrast to our rat study, which revealed differences in the metabolic excretion profiles between male and female animals (Kalisch et al. 2024), Bohlen et al. (2024) found no gender differences in GSH-BDA excretion rates in humans but observed significant differences in excretion rates established at different time-points. As the excretion of GSH-BDA in rats and humans is low, minor differences in the excretion rates used to calculate PDIs may well impact human exposure estimates. Human toxicokinetic studies using ^13^C-labeled furan may provide more accurate data on GSH-BDA excretion rates in humans and thus more accurate measures of human exposure to furan.

In our study, one individual (male, non-smoker) consistently exhibited significantly higher excretion of GSH-BDA compared to the remaining participants, while urinary excretion of NAcLys-BDA, NAcCys-BDA-Lys and NAcCys-BDA-Lys sulfoxide was within the range of levels observed in non-smoking subjects. The nutrition diary and health status of this individual provided no indication for diet, lifestyle or medication as plausible sources for these unexpectedly high GSH-BDA levels. While the origin of the high urinary GSH-BDA excretion observed in this subject thus remains unclear, these findings emphasize the importance of addressing interindividual differences in biomarker excretion and clarification of possible exogenous and endogenous sources of exposure. The large interindividual difference between GSH-BDA excretion in this individual compared to the remaining study participants currently presents a limitation of the use of GSH-BDA as a biomarker that warrants further investigation, including data on the overall variability of GSH-BDA excretion in a larger population.

Overall, the available data from rats and humans show that GSH-BDA is rapidly excreted in urine within 24 h of furan intake and correlates with furan dose. Despite the low excretion rates, analysis of GSH-BDA in our study provided estimates of furan exposure that are in line with the latest exposure assessment conducted by EFSA (2017), supporting GSH-BDA as a reliable biomarker of furan exposure. However, our data also indicate smoking as an important non-dietary exogenous source of furan exposure and contributor to urinary excretion of GSH-BDA in humans.

As a product of incomplete combustion, furan is well established to occur in mainstream tobacco smoke at concentrations 7–65 μg/cigarette. Smoking is thus recognized as a non-dietary source of furan exposure. Although information on exposure, bioavailability and toxicokinetics of furan after inhalation exposure are lacking, there has been some interest to investigate furan metabolites as biomarkers of furan inhalation exposure via cigarette smoke. Previous studies investigating the impact of smoking on the excretion of furan metabolites focused on BDA-derived lysine adducts and cysteine-lysine crosslinks and reported significantly increased concentrations of NAcCys-BDA-Lys and NAcCys-BDA-Lys sulfoxide in the urine of tobacco smokers relative to non-smokers (Grill et al. 2015; Vevang et al. 2023). In contrast, urinary levels of NAcLys-BDA, the most abundant furan metabolite in human urine, did not differ significantly between smokers and non-smokers. This is consistent with our data which also indicate that–in contrast to GSH-BDA, NAcCys-BDA-Lys and NAcCys-BDA-Lys sulfoxide—urinary excretion of NAcLys-BDA is unaffected by tobacco smoke exposure. Vevang et al. (2023) hypothesized that NAcLys-BDA may be formed primarily by hepatic biotransformation of furan after oral intake, whereas NAcCys-BDA-Lys and NAcCys-BDA-Lys sulfoxide were suggested to derive from extrahepatic metabolism of furan following inhalation exposure, presumably due to the abundance of CYP2E1 in pulmonary Clara cells, the target cells of furan inhalation toxicity (Tǎbǎran et al. 2019). Inhalation studies in mice appear to confirm preferential formation of cross-links between lysine and cysteine vs. direct reaction of BDA with lysine following inhalation exposure to furan (Tǎbǎran et al. 2019). However, since all furan metabolites are formed subsequent to CYP2E1-mediated bioactivation of furan to the reactive BDA, the only plausible explanation for differences in the metabolite profile resulting from hepatic vs. extrahepatic furan metabolism may be interorgan differences in the concentration of tissue nucleophiles such as GSH and lysine. However, there are at present no data to support this.

Of note, urinary concentrations of NAcLys-BDA, NAcCys-BDA-Lys and NAcCys-BDA-Lys sulfoxide in non-smokers and smokers were in the same range across studies reported by Grill et al. (2015). NAcLys-BDA was also consistently identified as the most abundant furan metabolite, with a similar concentration range across study cohorts. For instance, NAcLys-BDA in urine samples of the Persistence of Biomarkers (POB) study cohort reported by Grill et al. (2015) were 420 ± 430 pmol/mg creatinine in non-smokers and 420 ± 280 pmol/mg creatinine in smokers. These NAcLys-BDA concentrations are comparable to those found in non-smoking and smoking individuals at the onset of our study, i.e. 373.6 ± 135.3 pmol/mg creatinine in non-smokers and 392.2 ± 187.6 pmol/mg creatinine in smokers. Kremer et al. (2023) reported excretion of NAcLys-BDA at 0.505 µmol/24 h in non-smokers following coffee consumption, which compares to 0.279 µmol/24 h during periods of low-furan diet and 0.780 µmol/24 h following high-furan diet in our study.

Although urinary excretion of NAcLys-BDA increased in response to a high furan diet in our study, which may indicate furan-rich diet as a source of exposure, Bohlen et al. (2024) did not observe increased excretion of NAcLys-BDA following intake of a defined amount of furan via consumption of coffee brew. More importantly, the daily amount of NAcLys-BDA excreted on the days of furan intake via coffee was considerably higher than the amount of furan delivered (e.g. in male volunteers—day 4: 959.5 nmol NAcLys-BDA vs. 230 nmol furan; day 8: 776.2 nmol NAcLys-BDA vs. 676 nmol furan). In the tightly controlled duplicate diet study, exposure to furan via food was negligible compared to the intake via coffee (< 2.5%). Collectively, these data indicate that NAcLys-BDA in urine does not primarily derive from furan in food, and suggest a further, yet unidentified and potentially dietary source to account for NAcLys-BDA excretion. Alternatively, the presence of BDA-derived lysine adducts and cysteine-lysine crosslinks in the urine of untreated rats and humans during periods of low-furan diet may point towards an endogenous source of these metabolites. Peroxidation of unsaturated fatty acids and 5’-oxidation of deoxyribose have previously been suggested as potential endogenous pathways of furan formation (Kalisch et al. 2024).

Taken together, results from this study provide proof of concept for the use of a biomarker-based approach to monitor furan exposure from food. Human biomonitoring data further confirm GSH-BDA as a suitable biomarker of furan exposure by providing realistic exposure estimates close to those reported by EFSA (2017). Defining relative excretion rates of GSH-BDA in humans, including potential gender differences, employing isotopically labeled furan may help further refine furan exposure assessment based on biomarker monitoring.

## Supplementary Information

Below is the link to the electronic supplementary material.

Supplementary file 1 (DOCX 66 kb)

## Data Availability

All data supporting the findings of this study are available within the paper and its Supplementary Information. Raw data are available from the corresponding author upon reasonable request.

## Abbreviations

BDA: *cis*-2-Butene-1,4-dial
GSH: Glutathione
NAcLys: N-Acetyl-lysine
NAcCys: N-Acetyl-cysteine
GSH-BDA: *N*-[4-Carboxy-4-(3-mercapto-1*H*-pyrrol-1-yl)-1-oxobutyl]-L-cysteinylglycine
NAcLys-BDA: *R*-2-(Acetylamino)-6-(2,5-dihydro-2-oxo-1*H*-pyrrol-1-yl)-1-hexanoic acid
NAcCys-BDA-NAcLys: *N*-Acetyl-S-[1-[5-(acetylamino)-5-carboxypentyl]-1*H*-pyrrol-3-yl]-L-cysteine
NAcCys-BDA-NAcLys sulfoxide: *N*-Acetyl-S-[1-[5-(acetylamino)-5-carboxypentyl]-1*H*-pyrrol-3-yl]-L-cysteine sulfoxide
ESI-LC–MS/MS: Electrospray ionization liquid chromatography tandem mass spectrometry
