# Supplementary material for: Biomarker-based approach to human exposure assessment of furan in food
Source: Arch Toxicol. 2025 Apr 4;99(7):2819–34. doi: 10.1007/s00204-025-04022-2 (PMC12198286; doi:10.1007/s00204-025-04022-2)
Supplement: Supplementary file 1 — (DOCX 66 kb) [file 204_2025_4022_MOESM1_ESM.docx]

**Supplementary material**

**Supplementary tables**

**Table S1:** Dietary specifications for low-furan and high-furan study segments.

| **Meal** | **Low-furan diet** | **High-furan diet** |
| --- | --- | --- |
| *Beverages* | Water  Tea (except black tea) | Coffee  Fruit juices |
| *Breakfast* | Oats (uncooked or cooked)  Milk and dairy products (e.g. cottage cheese, plain yoghurt, feta cheese)  Fresh fruits and vegetables  Boiled eggs | Granola, puffed cereals (e.g. Honey wheat)  Dark toasted white bread  Canned or jarred spreads |
| *Lunch and dinner* | Steamed or boiled vegetables  Steamed or boiled fish or meat  Rice, potatoes, noodles  Boiled eggs  Salad with dressing (vinegar, oil)  Seasonings: fresh herbs, lemon juice, salt, fresh garlic or onion | Canned or jarred foods:   - Stews (e.g. chili con carne, lentil stew) - Soups - Pasta - Sauces (e.g. tomato sauce) - Baked Beans - Processed baby and infant food   Pizza  French fries (potato, sweet potato)  Seasonings (e.g. soy sauce, Worcester sauce, ketchup, tomato paste) |
| *Snacks* | Fresh fruits and vegetables  Nuts (non-roasted) | Canned fruits and vegetables  Roasted nuts  Crackers (rice or corn)  Popcorn  Potato chips  Cookies  Caramel |

**Table S2:** Probable daily furan intakes (µg/kg bw/d) of non-smoking (n = 6) and smoking subjects (n = 4) for different diet types based on urinary metabolite excretion and relative excretion rates of the respective metabolites, previously determined in male and female F344/DuCrl rats (Kalisch et al., 2024). Data are presented as mean (± SD) and range (min. – max.). Subject 2 (male, non-smoker) was excluded for analysis of PDIs based on urinary GSH-BDA excretion.

| **Metabolite** | **Diet type** | **Probable daily furan intakes (µg/kg bw/d)** | | | |
| --- | --- | --- | --- | --- | --- |
|  |  | **Based on relative excretion rate determined in male rats^1^** | | **Based on relative excretion rates determined in female rats^2^** | |
|  |  | **mean (± SD)** | **min. – max.** | **mean (± SD)** | **min. – max.** |
| *Non-smoking subjects* | | | | | |
| GSH-BDA | normal | 0.33 ± 0.35 | 0.05 – 0.90 | 0.60 ± 0.65 | 0.09 – 1.67 |
|  | low-furan | 0.12 ± 0.06 | 0.05 – 0.31 | 0.22 ± 0.10 | 0.09 – 0.57 |
|  | high-furan | 0.63 ± 0.35 | 0.18 – 1.20 | 1.16 ± 0.65 | 0.32 – 2.22 |
| NAcLys-BDA | normal | 113.5 ± 48.1 | 41.0 – 182.4 | 128.7 ± 54.6 | 46.5 – 207.0 |
|  | low-furan | 45.3 ± 18.3 | 17.9 – 96.4 | 51.4 ± 20.8 | 20.3 – 109.4 |
|  | high-furan | 126.2 ± 31.9 | 85.4 – 204.0 | 143.2 ± 36.2 | 96.9 – 231.5 |
| NAcCys-BDA-Lys | normal | 1.94 ± 0.71 | 0.98 – 2.70 | 1.43 ± 0.53 | 0.72 – 1.99 |
|  | low-furan | 1.86 ± 0.67 | 0.82 – 3.84 | 1.37 ± 0.49 | 0.61 – 2.83 |
|  | high-furan | 3.13 ± 1.13 | 1.32 – 5.47 | 2.31 ± 0.83 | 0.98 – 4.04 |
| NAcCys-BDA-Lys sulfoxide | normal | 2.00 ± 0.94 | 0.62 – 3.11 | 4.92 ± 2.31 | 1.54 – 7.66 |
|  | low-furan | 1.25 ± 1.01 | 0.17 – 3.94 | 3.07 ± 2.49 | 0.42 – 9.70 |
|  | high-furan | 1.92 ± 0.91 | 0.20 – 3.38 | 4.74 ± 2.24 | 0.49 – 8.32 |
| *Smoking subjects* | | | | | |
| GSH-BDA | normal | 1.12 ± 0.74 | 0.47 – 2.00 | 2.07 ± 1.36 | 0.88 – 2.71 |
|  | low-furan | 0.81 ± 0.54 | 0.13 – 1.67 | 1.49 ± 0.99 | 0.23 – 3.09 |
|  | high-furan | 1.64 ± 0.71 | 0.52 – 2.70 | 3.03 ± 1.32 | 0.97 – 4.98 |
| NAcLys-BDA | normal | 120.2 ± 58.4 | 47.6 – 174.6 | 136.4 ± 66.3 | 54.0 – 198.1 |
|  | low-furan | 56.3 ± 25.9 | 22.8 – 109.0 | 63.9 ± 29.4 | 25.9 – 123.7 |
|  | high-furan | 135.3 ± 65.0 | 72.6 – 263.5 | 153.5 ± 73.8 | 82.3 – 299.0 |
| NAcCys-BDA-Lys | normal | 2.44 ± 1.43 | 1.06 – 4.04 | 1.80 ± 1.05 | 0.78 – 2.98 |
|  | low-furan | 2.72 ± 1.58 | 0.93 – 5.42 | 2.01 ± 1.16 | 0.69 – 4.01 |
|  | high-furan | 5.02 ± 2.88 | 1.92 – 9.86 | 3.70 ± 2.12 | 1.41 – 7.28 |
| NAcCys-BDA-Lys sulfoxide | normal | 2.77 ± 2.58 | 0.46 – 6.37 | 6.82 ± 6.35 | 1.14 – 15.7 |
|  | low-furan | 2.78 ± 2.17 | 0.31 – 7.09 | 6.85 ± 5.34 | 0.77 – 17.5 |
|  | high-furan | 3.51 ± 3.12 | 0.34 – 8.05 | 8.66 ± 7.68 | 0.83 – 19.8 |

^1^ Relative excretion rates determined in male F344/DuCrl: GSH-BDA (2.18 %), NAcLys-BDA (0.59 %), NAcCys-BDA-Lys (1.58 %), NAcCys-BDA-Lys sulfoxide (0.69 %)

^2^ Relative excretion rates determined in female F344/DuCrl: GSH-BDA (1.18 %), NAcLys-BDA (0.52 %), NAcCys-BDA-Lys (2.14 %), NAcCys-BDA-Lys sulfoxide (0.28 %)

**Table S3:** Calculation of probable daily intakes (PDI) for smoking subjects based on urinary GSH-BDA excretion and GSH-BDA excretion rate determined in male rats (2.18 %).

| **Day** | **Subject** | **Absolute excretion (µg/24 h)** | **Absolute excretion (nmol/24 h)** | **Probable furan dose (nmol/24 h)** | **Probable furan dose (µg/24 h)** | **Body weight (kg)** | **Probable daily intake (µg/kg bw/d)** | | |
| --- | --- | --- | --- | --- | --- | --- | --- | --- | --- |
|  |  |  |  |  |  |  |  | **Mean** | **SD** |
| 0 | 1 | 4.59 | 12.93 | 593.2 | 40.34 | 85 | 0.47 | 1.12 | 0.74 |
|  | 5 | 19.83 | 55.85 | 2562.0 | 174.22 | 87.3 | 2.00 |  |  |
|  | 7 | 3.98 | 11.22 | 514.7 | 35.00 | 63 | 0.56 |  |  |
|  | 10 | 13.02 | 36.68 | 1682.6 | 114.41 | 78 | 1.47 |  |  |
| 1 | 1 | 4.30 | 12.11 | 555.7 | 37.79 | 85 | 0.44 | 0.92 | 0.49 |
|  | 5 | 14.34 | 40.40 | 1853.1 | 126.01 | 87.3 | 1.44 |  |  |
|  | 7 | 4.02 | 11.33 | 519.7 | 35.34 | 63 | 0.56 |  |  |
|  | 10 | 10.90 | 30.70 | 1408.1 | 95.75 | 78 | 1.23 |  |  |
| 2 | 1 | 2.08 | 5.87 | 269.3 | 18.31 | 85 | 0.22 | 0.71 | 0.52 |
|  | 5 | 11.26 | 31.73 | 1455.5 | 98.98 | 87.3 | 1.13 |  |  |
|  | 7 | 2.13 | 6.01 | 275.8 | 18.75 | 63 | 0.30 |  |  |
|  | 10 | 10.58 | 29.79 | 1366.6 | 92.93 | 78 | 1.19 |  |  |
| 3 | 1 | 1.31 | 3.68 | 168.9 | 11.48 | 85 | 0.14 | 0.89 | 0.75 |
|  | 5 | 16.60 | 46.77 | 2145.6 | 145.90 | 87.3 | 1.67 |  |  |
|  | 7 | 2.69 | 7.58 | 347.5 | 23.63 | 63 | 0.38 |  |  |
|  | 10 | 12.24 | 34.48 | 1581.8 | 107.56 | 78 | 1.38 |  |  |
| 4 | 1 | 5.07 | 14.28 | 655.0 | 44.54 | 85 | 0.52 | 1.34 | 0.62 |
|  | 5 | 14.87 | 41.88 | 1921.1 | 130.63 | 87.3 | 1.50 |  |  |
|  | 7 | 9.46 | 26.65 | 1222.3 | 83.11 | 63 | 1.32 |  |  |
|  | 10 | 18.03 | 50.79 | 2329.7 | 158.42 | 78 | 2.03 |  |  |
| 5 | 1 | 6.93 | 19.52 | 895.5 | 60.89 | 85 | 0.72 | 1.91 | 0.91 |
|  | 5 | 26.79 | 75.47 | 3462.0 | 235.42 | 87.3 | 2.70 |  |  |
|  | 7 | 12.19 | 34.34 | 1575.4 | 107.13 | 63 | 1.70 |  |  |
|  | 10 | 22.48 | 63.32 | 2904.6 | 197.51 | 78 | 2.53 |  |  |
| 6 | 1 | 7.52 | 21.19 | 972.1 | 66.10 | 85 | 0.78 | 1.66 | 0.66 |
|  | 5 | 18.56 | 52.29 | 2398.8 | 163.12 | 87.3 | 1.87 |  |  |
|  | 7 | 11.67 | 32.87 | 1507.8 | 102.53 | 63 | 1.63 |  |  |
|  | 10 | 21.02 | 59.20 | 2715.5 | 184.65 | 78 | 2.37 |  |  |
| 7 | 1 | 1.58 | 4.45 | 204.2 | 13.88 | 85 | 0.16 | 0.87 | 0.68 |
|  | 5 | 12.45 | 35.07 | 1608.9 | 109.40 | 87.3 | 1.25 |  |  |
|  | 7 | 3.23 | 9.09 | 417.1 | 28.37 | 63 | 0.45 |  |  |
|  | 10 | 14.44 | 40.69 | 1866.3 | 126.91 | 78 | 1.63 |  |  |
| 8 | 1 | 3.66 | 10.32 | 473.5 | 32.20 | 85 | 0.38 | 0.79 | 0.50 |
|  | 5 | 9.72 | 27.37 | 1255.5 | 85.38 | 87.3 | 0.98 |  |  |
|  | 7 | 2.72 | 7.65 | 350.9 | 23.86 | 63 | 0.38 |  |  |
|  | 10 | 12.47 | 35.13 | 1611.6 | 109.59 | 78 | 1.41 |  |  |
| 9 | 1 | 1.23 | 3.45 | 158.3 | 10.76 | 85 | 0.13 | 0.68 | 0.58 |
|  | 5 | 12.37 | 34.83 | 1597.8 | 108.65 | 87.3 | 1.24 |  |  |
|  | 7 | 1.60 | 4.49 | 206.1 | 14.01 | 63 | 0.22 |  |  |
|  | 10 | 9.90 | 27.88 | 1279.1 | 86.98 | 78 | 1.12 |  |  |

**Table S4:** Calculation of probable daily intakes (PDI) for non-smoking subjects based on urinary GSH-BDA excretion and GSH-BDA excretion rate determined in male rats (2.18 %).

| **Day** | **Subject** | **Absolute excretion (µg/24 h)** | **Absolute excretion (nmol/24 h)** | **Probable furan dose (nmol/24 h)** | **Probable furan dose (µg/24 h)** | **Body weight (kg)** | **Probable daily intake (µg/kg bw/d)** | | |
| --- | --- | --- | --- | --- | --- | --- | --- | --- | --- |
|  |  |  |  |  |  |  |  | **Mean** | **SD** |
| 0 | 3 | 0.41 | 1.15 | 52.7 | 3.58 | 71.7 | 0.05 | 0.33 | 0.35 |
|  | 4 | 3.94 | 11.10 | 509.3 | 34.64 | 80 | 0.43 |  |  |
|  | 6 | 0.88 | 2.49 | 114.3 | 7.77 | 63 | 0.12 |  |  |
|  | 8 | 0.78 | 2.20 | 100.8 | 6.85 | 55 | 0.12 |  |  |
|  | 9 | 7.22 | 20.33 | 932.4 | 63.40 | 70.2 | 0.90 |  |  |
| 1 | 3 | 0.44 | 1.25 | 57.1 | 3.88 | 71.7 | 0.05 | 0.14 | 0.06 |
|  | 4 | 2.03 | 5.72 | 262.3 | 17.84 | 80 | 0.22 |  |  |
|  | 6 | 0.94 | 2.66 | 122.0 | 8.29 | 63 | 0.13 |  |  |
|  | 8 | 0.72 | 2.04 | 93.6 | 6.36 | 55 | 0.12 |  |  |
|  | 9 | 1.24 | 3.48 | 159.6 | 10.85 | 70.2 | 0.15 |  |  |
| 2 | 3 | 0.52 | 1.48 | 67.7 | 4.60 | 71.7 | 0.06 | 0.11 | 0.03 |
|  | 4 | 1.30 | 3.67 | 168.2 | 11.44 | 80 | 0.14 |  |  |
|  | 6 | 0.77 | 2.18 | 100.0 | 6.80 | 63 | 0.11 |  |  |
|  | 8 | 0.83 | 2.34 | 107.5 | 7.31 | 55 | 0.13 |  |  |
|  | 9 | 0.77 | 2.18 | 100.0 | 6.80 | 70.2 | 0.10 |  |  |
| 3 | 3 | 0.45 | 1.26 | 57.6 | 3.92 | 71.7 | 0.05 | 0.10 | 0.03 |
|  | 4 | 1.07 | 3.02 | 138.5 | 9.42 | 80 | 0.12 |  |  |
|  | 6 | 0.89 | 2.51 | 115.0 | 7.82 | 63 | 0.12 |  |  |
|  | 8 | 0.64 | 1.81 | 83.2 | 5.66 | 55 | 0.10 |  |  |
|  | 9 | 0.68 | 1.93 | 88.4 | 6.01 | 70.2 | 0.09 |  |  |
| 4 | 3 | 2.70 | 7.62 | 349.5 | 23.77 | 71.7 | 0.33 | 0.51 | 0.30 |
|  | 4 | 2.76 | 7.76 | 356.0 | 24.21 | 80 | 0.30 |  |  |
|  | 6 | 5.27 | 14.83 | 680.4 | 46.27 | 63 | 0.73 |  |  |
|  | 8 | 1.58 | 4.44 | 203.5 | 13.84 | 55 | 0.25 |  |  |
|  | 9 | 7.38 | 20.79 | 953.7 | 64.85 | 70.2 | 0.92 |  |  |
| 5 | 3 | 2.41 | 6.80 | 312.0 | 21.21 | 71.7 | 0.30 | 0.68 | 0.38 |
|  | 4 | 5.85 | 16.48 | 756.1 | 51.41 | 80 | 0.64 |  |  |
|  | 6 | 6.51 | 18.33 | 840.8 | 57.18 | 63 | 0.91 |  |  |
|  | 8 | 2.28 | 6.41 | 294.1 | 20.00 | 55 | 0.36 |  |  |
|  | 9 | 9.60 | 27.03 | 1239.9 | 84.31 | 70.2 | 1.20 |  |  |
| 6 | 3 | 1.44 | 4.04 | 185.4 | 12.61 | 71.7 | 0.18 | 0.69 | 0.42 |
|  | 4 | 4.46 | 12.55 | 575.8 | 39.16 | 80 | 0.49 |  |  |
|  | 6 | 7.53 | 21.22 | 973.6 | 66.20 | 63 | 1.05 |  |  |
|  | 8 | 3.35 | 9.44 | 432.8 | 29.43 | 55 | 0.54 |  |  |
|  | 9 | 9.41 | 26.49 | 1215.3 | 82.64 | 70.2 | 1.18 |  |  |
| 7 | 3 | 0.93 | 2.63 | 120.4 | 8.19 | 71.7 | 0.11 | 0.19 | 0.08 |
|  | 4 | 1.15 | 3.23 | 148.3 | 10.09 | 80 | 0.13 |  |  |
|  | 6 | 1.68 | 4.73 | 216.8 | 14.74 | 63 | 0.23 |  |  |
|  | 8 | 0.99 | 2.80 | 128.4 | 8.73 | 55 | 0.16 |  |  |
|  | 9 | 2.48 | 6.97 | 319.8 | 21.75 | 70.2 | 0.31 |  |  |
| 8 | 3 | 0.40 | 1.13 | 51.9 | 3.53 | 71.7 | 0.05 | 0.09 | 0.03 |
|  | 4 | 1.01 | 2.86 | 131.0 | 8.91 | 80 | 0.11 |  |  |
|  | 6 | 0.80 | 2.26 | 103.9 | 7.06 | 63 | 0.11 |  |  |
|  | 8 | 0.64 | 1.81 | 83.0 | 5.64 | 55 | 0.10 |  |  |
|  | 9 | 0.58 | 1.65 | 75.5 | 5.13 | 70.2 | 0.07 |  |  |
| 9 | 3 | 0.44 | 1.25 | 57.4 | 3.90 | 71.7 | 0.05 | 0.10 | 0.03 |
|  | 4 | 1.10 | 3.10 | 142.4 | 9.68 | 80 | 0.12 |  |  |
|  | 6 | 0.82 | 2.30 | 105.7 | 7.19 | 63 | 0.11 |  |  |
|  | 8 | 0.66 | 1.86 | 85.3 | 5.80 | 55 | 0.11 |  |  |
|  | 9 | 0.92 | 2.60 | 119.4 | 8.12 | 70.2 | 0.12 |  |  |

**Table S5:** Calculation of probable daily intakes (PDI) for smoking subjects based on urinary GSH-BDA excretion and GSH-BDA excretion rate determined in female rats (1.18 %).

| **Day** | **Subject** | **Absolute excretion (µg/24 h)** | **Absolute excretion (nmol/24 h)** | **Probable furan dose (nmol/24 h)** | **Probable furan dose (µg/24 h)** | **Body weight (kg)** | **Probable daily intake (µg/kg bw/d)** | | |
| --- | --- | --- | --- | --- | --- | --- | --- | --- | --- |
|  |  |  |  |  |  |  |  | **Mean** | **SD** |
| 0 | 1 | 4.59 | 12.93 | 1095.9 | 74.52 | 85 | 0.88 | 2.07 | 1.36 |
|  | 5 | 19.83 | 55.85 | 4733.2 | 321.86 | 87.3 | 3.69 |  |  |
|  | 7 | 3.98 | 11.22 | 950.9 | 64.66 | 63 | 1.03 |  |  |
|  | 10 | 13.02 | 36.68 | 3108.5 | 211.38 | 78 | 2.71 |  |  |
| 1 | 1 | 4.30 | 12.11 | 1026.6 | 69.81 | 85 | 0.82 | 1.70 | 0.91 |
|  | 5 | 14.34 | 40.40 | 3423.5 | 232.80 | 87.3 | 2.67 |  |  |
|  | 7 | 4.02 | 11.33 | 960.2 | 65.29 | 63 | 1.04 |  |  |
|  | 10 | 10.90 | 30.70 | 2601.5 | 176.90 | 78 | 2.27 |  |  |
| 2 | 1 | 2.08 | 5.87 | 497.5 | 33.83 | 85 | 0.40 | 1.31 | 0.97 |
|  | 5 | 11.26 | 31.73 | 2689.1 | 182.86 | 87.3 | 2.09 |  |  |
|  | 7 | 2.13 | 6.01 | 509.5 | 34.65 | 63 | 0.55 |  |  |
|  | 10 | 10.58 | 29.79 | 2524.7 | 171.68 | 78 | 2.20 |  |  |
| 3 | 1 | 1.31 | 3.68 | 312.0 | 21.22 | 85 | 0.25 | 1.64 | 1.38 |
|  | 5 | 16.60 | 46.77 | 3963.8 | 269.54 | 87.3 | 3.09 |  |  |
|  | 7 | 2.69 | 7.58 | 642.0 | 43.66 | 63 | 0.69 |  |  |
|  | 10 | 12.24 | 34.48 | 2922.2 | 198.71 | 78 | 2.55 |  |  |
| 4 | 1 | 5.07 | 14.28 | 1210.2 | 82.29 | 85 | 0.97 | 2.48 | 1.15 |
|  | 5 | 14.87 | 41.88 | 3549.1 | 241.34 | 87.3 | 2.76 |  |  |
|  | 7 | 9.46 | 26.65 | 2258.1 | 153.55 | 63 | 2.44 |  |  |
|  | 10 | 18.03 | 50.79 | 4304.0 | 292.67 | 78 | 3.75 |  |  |
| 5 | 1 | 6.93 | 19.52 | 1654.4 | 112.50 | 85 | 1.32 | 3.53 | 1.68 |
|  | 5 | 26.79 | 75.47 | 6395.9 | 434.92 | 87.3 | 4.98 |  |  |
|  | 7 | 12.19 | 34.34 | 2910.5 | 197.91 | 63 | 3.14 |  |  |
|  | 10 | 22.48 | 63.32 | 5366.1 | 364.90 | 78 | 4.68 |  |  |
| 6 | 1 | 7.52 | 21.19 | 1795.9 | 122.12 | 85 | 1.44 | 3.07 | 1.23 |
|  | 5 | 18.56 | 52.29 | 4431.6 | 301.35 | 87.3 | 3.45 |  |  |
|  | 7 | 11.67 | 32.87 | 2785.6 | 189.42 | 63 | 3.01 |  |  |
|  | 10 | 21.02 | 59.20 | 5016.7 | 341.14 | 78 | 4.37 |  |  |
| 7 | 1 | 1.58 | 4.45 | 377.2 | 25.65 | 85 | 0.30 | 1.61 | 1.26 |
|  | 5 | 12.45 | 35.07 | 2972.3 | 202.12 | 87.3 | 2.32 |  |  |
|  | 7 | 3.23 | 9.09 | 770.6 | 52.40 | 63 | 0.83 |  |  |
|  | 10 | 14.44 | 40.69 | 3447.9 | 234.46 | 78 | 3.01 |  |  |
| 8 | 1 | 3.66 | 10.32 | 874.7 | 59.48 | 85 | 0.70 | 1.45 | 0.92 |
|  | 5 | 9.72 | 27.37 | 2319.5 | 157.73 | 87.3 | 1.81 |  |  |
|  | 7 | 2.72 | 7.65 | 648.3 | 44.08 | 63 | 0.70 |  |  |
|  | 10 | 12.47 | 35.13 | 2977.4 | 202.46 | 78 | 2.60 |  |  |
| 9 | 1 | 1.23 | 3.45 | 292.4 | 19.89 | 85 | 0.23 | 1.25 | 1.08 |
|  | 5 | 12.37 | 34.83 | 2951.9 | 200.73 | 87.3 | 2.30 |  |  |
|  | 7 | 1.60 | 4.49 | 380.8 | 25.89 | 63 | 0.41 |  |  |
|  | 10 | 9.90 | 27.88 | 2363.0 | 160.69 | 78 | 2.06 |  |  |

**Table S6:** Calculation of probable daily intakes (PDI) for non-smoking subjects based on urinary GSH-BDA excretion and GSH-BDA excretion rate determined in female rats (1.18 %).

| **Day** | **Subject** | **Absolute excretion (µg/24 h)** | **Absolute excretion (nmol/24 h)** | **Probable furan dose (nmol/24 h)** | **Probable furan dose (µg/24 h)** | **Body weight (kg)** | **Probable daily intake (µg/kg bw/d)** | | |
| --- | --- | --- | --- | --- | --- | --- | --- | --- | --- |
|  |  |  |  |  |  |  |  | **Mean** | **SD** |
| 0 | 3 | 0.41 | 1.15 | 97.4 | 6.62 | 71.7 | 0.09 | 0.60 | 0.65 |
|  | 4 | 3.94 | 11.10 | 941.0 | 63.99 | 80 | 0.80 |  |  |
|  | 6 | 0.88 | 2.49 | 211.1 | 14.36 | 63 | 0.23 |  |  |
|  | 8 | 0.78 | 2.20 | 186.2 | 12.66 | 55 | 0.23 |  |  |
|  | 9 | 7.22 | 20.33 | 1722.5 | 117.13 | 70.2 | 1.67 |  |  |
| 1 | 3 | 0.44 | 1.25 | 105.5 | 7.17 | 71.7 | 0.10 | 0.25 | 0.11 |
|  | 4 | 2.03 | 5.72 | 484.6 | 32.95 | 80 | 0.41 |  |  |
|  | 6 | 0.94 | 2.66 | 225.4 | 15.32 | 63 | 0.24 |  |  |
|  | 8 | 0.72 | 2.04 | 172.8 | 11.75 | 55 | 0.21 |  |  |
|  | 9 | 1.24 | 3.48 | 294.8 | 20.05 | 70.2 | 0.29 |  |  |
| 2 | 3 | 0.52 | 1.48 | 125.1 | 8.51 | 71.7 | 0.12 | 0.20 | 0.06 |
|  | 4 | 1.30 | 3.67 | 310.8 | 21.14 | 80 | 0.26 |  |  |
|  | 6 | 0.77 | 2.18 | 184.8 | 12.56 | 63 | 0.20 |  |  |
|  | 8 | 0.83 | 2.34 | 198.6 | 13.51 | 55 | 0.25 |  |  |
|  | 9 | 0.77 | 2.18 | 184.8 | 12.56 | 70.2 | 0.18 |  |  |
| 3 | 3 | 0.45 | 1.26 | 106.5 | 7.24 | 71.7 | 0.10 | 0.18 | 0.05 |
|  | 4 | 1.07 | 3.02 | 255.9 | 17.40 | 80 | 0.22 |  |  |
|  | 6 | 0.89 | 2.51 | 212.5 | 14.45 | 63 | 0.23 |  |  |
|  | 8 | 0.64 | 1.81 | 153.7 | 10.45 | 55 | 0.19 |  |  |
|  | 9 | 0.68 | 1.93 | 163.3 | 11.10 | 70.2 | 0.16 |  |  |
| 4 | 3 | 2.70 | 7.62 | 645.7 | 43.91 | 71.7 | 0.61 | 0.94 | 0.56 |
|  | 4 | 2.76 | 7.76 | 657.7 | 44.72 | 80 | 0.56 |  |  |
|  | 6 | 5.27 | 14.83 | 1257.0 | 85.48 | 63 | 1.36 |  |  |
|  | 8 | 1.58 | 4.44 | 376.0 | 25.57 | 55 | 0.46 |  |  |
|  | 9 | 7.38 | 20.79 | 1762.0 | 119.82 | 70.2 | 1.71 |  |  |
| 5 | 3 | 2.41 | 6.80 | 576.4 | 39.19 | 71.7 | 0.55 | 1.26 | 0.70 |
|  | 4 | 5.85 | 16.48 | 1396.8 | 94.98 | 80 | 1.19 |  |  |
|  | 6 | 6.51 | 18.33 | 1553.4 | 105.63 | 63 | 1.68 |  |  |
|  | 8 | 2.28 | 6.41 | 543.3 | 36.95 | 55 | 0.67 |  |  |
|  | 9 | 9.60 | 27.03 | 2290.6 | 155.76 | 70.2 | 2.22 |  |  |
| 6 | 3 | 1.44 | 4.04 | 342.6 | 23.29 | 71.7 | 0.32 | 1.27 | 0.77 |
|  | 4 | 4.46 | 12.55 | 1063.8 | 72.34 | 80 | 0.90 |  |  |
|  | 6 | 7.53 | 21.22 | 1798.6 | 122.31 | 63 | 1.94 |  |  |
|  | 8 | 3.35 | 9.44 | 799.6 | 54.37 | 55 | 0.99 |  |  |
|  | 9 | 9.41 | 26.49 | 2245.2 | 152.67 | 70.2 | 2.17 |  |  |
| 7 | 3 | 0.93 | 2.63 | 222.5 | 15.13 | 71.7 | 0.21 | 0.35 | 0.15 |
|  | 4 | 1.15 | 3.23 | 274.1 | 18.64 | 80 | 0.23 |  |  |
|  | 6 | 1.68 | 4.73 | 400.6 | 27.24 | 63 | 0.43 |  |  |
|  | 8 | 0.99 | 2.80 | 237.3 | 16.14 | 55 | 0.29 |  |  |
|  | 9 | 2.48 | 6.97 | 590.9 | 40.18 | 70.2 | 0.57 |  |  |
| 8 | 3 | 0.40 | 1.13 | 96.0 | 6.53 | 71.7 | 0.09 | 0.17 | 0.05 |
|  | 4 | 1.01 | 2.86 | 242.1 | 16.46 | 80 | 0.21 |  |  |
|  | 6 | 0.80 | 2.26 | 191.9 | 13.05 | 63 | 0.21 |  |  |
|  | 8 | 0.64 | 1.81 | 153.3 | 10.42 | 55 | 0.19 |  |  |
|  | 9 | 0.58 | 1.65 | 139.4 | 9.48 | 70.2 | 0.14 |  |  |
| 9 | 3 | 0.44 | 1.25 | 106.0 | 7.21 | 71.7 | 0.10 | 0.19 | 0.05 |
|  | 4 | 1.10 | 3.10 | 263.1 | 17.89 | 80 | 0.22 |  |  |
|  | 6 | 0.82 | 2.30 | 195.3 | 13.28 | 63 | 0.21 |  |  |
|  | 8 | 0.66 | 1.86 | 157.6 | 10.71 | 55 | 0.19 |  |  |
|  | 9 | 0.92 | 2.60 | 220.6 | 15.00 | 70.2 | 0.21 |  |  |
